# Supplementary material for: Asymmetric conformations and lipid interactions shape the ATP-coupled cycle of a heterodimeric ABC transporter
Source: Nat Commun. 2023 Nov 8;14:7184. doi: 10.1038/s41467-023-42937-5 (PMC10632425; doi:10.1038/s41467-023-42937-5)
Supplement: Supplementary file 1 — Supplementary information [file 41467_2023_42937_MOESM1_ESM.pdf]

Asymmetric conformations and lipid interactions shape the ATP-coupled cycle of a heterodimeric ABC transporter

Qingyu Tang[1], Matt Sinclair[2, #], Hale S. Hasdemir [2, #], Richard A. Stein[1], Erkan Karakas[1], Emad Tajkhorshid[2], & Hassane S. Mchaourab[1*]

[1] Department of Molecular Physiology and Biophysics, Vanderbilt University, Nashville, TN 37232, USA. [2]Theoretical and Computational Biophysics Group, NIH Resource for Macromolecular Modeling and Visualization, Beckman Institute for Advanced Science and Technology, Department of Biochemistry, and Center for Biophysics and Quantitative Biology, University of Illinois at Urbana-Champaign, Urbana, Illinois 61801, USA. [#] Contributed equally.
*email: hassane.mchaourab@vanderbilt.edu

Supplementary Table 1: Cryo-EM data collection, refinement, and validation statistics

**Data collection and processing**							
Dataset	Dataset 1	Dataset 2	Dataset 3	Dataset 4		Dataset 5	
Construct	BmrCD*-QQ	BmrCD*-QQ	BmrCD*-QQ	BmrCD*-QQ		BmrCD-WT	
Lipids used	PC/PA	PC/*E.coli* polar lipid	PC/PA	PC/*E.coli* polar lipid		PC/PA	
Supplemented ligands	ATP, Mg^{2+}, Hoechst	ATP, Mg^{2+}, Hoechst	ATP, Mg^{2+}	ATP, Mg^{2+}		ATP, Mg^{2+}, Hoechst, Vanadate	
Conformational state	**BmrCD_IF-2HT/ATP**	**BmrCD_IF-1HT/ATP**	**BmrCD_IF-ATP**	**BmrCD_IF-ATP2**	**BmrCD_OC-ATP**	**BmrCD_OC-ADPVi**	**BmrCD_IF-HT/ADPVi**
PDB ID	8FMV	8SZC	8FPF	8T3K	8FHK	8T1P	**
EMDB ID	EMD-29297	EMD-40908	EMD-29362	EMD-41004	EMD-29087	EMD-40974	EMD-41058
Symmetry imposed	C1	C1	C1	C1		C1	
Microscope	Titan Krios (FEI)	Titan Krios (FEI)	Titan Krios (FEI)	Titan Krios (FEI)		Titan Krios (FEI)	
Detector	Gatan K3	Gatan K3	Gatan K3	Gatan K3		Gatan K3	
Nominal magnification	105,000 x	105,000 x	81,000 x	130,000 x		130,000 x	
Voltage (kV)	300	300	300	300		300	
Electron exposure (e/Å^2)	48	51	54	52		56	
Defocus range (μm)	-0.8 to -2.2	-0.8 to -2.2	-0.8 to -1.6	-0.4 to -2.2		-0.9 to -2.0	
Pixel size (Å)	0.818	0.818	1.1	0.647		0.647	
Number of Micrographs	5,696	12,706	3,722	11,829		11,980	
Particles images	2,461,748	4,586,278	2,429,885	2,276,125	4,811,558	6,565,035	6,824,865
Final particles images	133,280	107,639	255,381	76,190	91,128	185,953	262,341
Map resolution (Å) (FSC threshold=0.143)	3.3	3.1	3.3	3.3	2.9	3.1	5.7
**Refinement**							
Model resolution (Å) (original map, FSC threshold=0.5)	3.6	3.7	3.8	7.1	3.8	3.2	
Model resolution (Å) (composite map, FSC threshold=0.5)	3.2	3.1	3.4	3.6	3.4	-*	
B-factor used for map sharpening (Å^2)	-104.6	-77.9	-112.4	-86.1	-90.7	-89.5	
Model composition							
Non-hydrogen atoms	10,501	10,511	10,624	10,144	10,120	10,587	
Protein residues	1236	1235	1,226	1,220	1,221	1,241	
ATP, Hoechst, lipids, Mg^{2+}, AOV	2, 2, 20, 1, 0	2, 1, 21, 1, 0	2, 0, 25, 0, 0	2, 0, 13, 0, 0	2, 0, 16, 2, 0	1, 0, 21, 2, 1	
Mean B factors (Å^2)							
Protein	78.26	70.63	79.69	62.42	61.64	59.73	
Ligands	75.14	72.92	63.71	50.26	60.04	77.21	
R.m.s. deviations							
Bond lengths (Å)	0.014	0.004	0.008	0.005	0.008	0.006	
Bond angles (°)	1.422	0.695	0.895	0.769	1.136	0.782	
Molprobity score	1.73	1.74	1.77	1.89	1.86	1.96	
Clash score	5.92	5.48	5.93	8.02	7.55	8.22	
Poor rotamers (%)	0.10	0.00	0.00	0.10	0.39	0.38	
Ramachandran plot							
Favored (%)	93.89	93.00	93.10	92.84	93.04	91.34	
Allowed (%)	6.11	7.00	6.90	7.16	6.96	8.66	
Disallowed (%)	0.00	0.00	0.00	0.00	0.00	0.00	

*: The model of BmrCD_OC-ADPVi was built by original map after Local refinement in CryoSPARC.
**: No structural model was built for BmrCD_IF-HT/ADPVi. The number of HT is not indicated.

Supplementary Table 2: Root mean square deviation (r.m.s.d.) of overall structure.

	BmrCD_det	BmrCD_IF-2HT/ATP	BmrCD_IF-1HT/ATP	BmrCD_IF-ATP	BmrCD_IF-ATP2	BmrCD_OC-ATP	BmrCD_OC-ADPVi
**BmrCD_det**	0.00	0.65 (959 Cα)	0.82 (992 Cα)	1.38 (1078 Cα)	0.92 (980 Cα)	6.24 (1190 Cα)	6.30 (1217 Cα)
**BmrCD_IF-2HT/ATP**		0.00	0.42 (1131 Cα)	0.94 (1153 Cα)	0.67 (1026 Cα)	6.48 (1199 Cα)	6.48 (1228 Cα)
**BmrCD_IF-1HT/ATP**			0.00	0.87 (1142 Cα)	0.75 (1031 Cα)	6.60 (1202 Cα)	6.58 (1229 Cα)
**BmrCD_IF-ATP**				0.00	0.99 (1096 Cα)	6.64 (1193 Cα)	6.62 (1219 Cα)
**BmrCD_IF-ATP2**					0.00	6.46 (1172 Cα)	6.40 (1194 Cα)
**BmrCD_OC-ATP**						0.00	0.63 (1039 Cα)
**BmrCD_OC-ADPVi**							0.00

Superposition was performed at the residue level by Pymol and r.m.s.d. was calculated at the Cα level in unit of Å.

Supplementary Table 3: Statistical values of angle space distribution for each surface basic residue for IF and Occluded conformations (For Fig. 7d).

			Occluded						IF					
**Residue**	**Monom**	**N**	**Mean**	**Std.**	**Min**	**25%**	**75%**	**Max**	**Mean**	**Std.**	**Min**	**25%**	**75%**	**Max**
6	BmrC	3000	49.61	8.25	23.59	43.91	54.67	78.65	49.90	6.30	23.28	45.71	53.95	76.66
7	BmrC	3000	144.76	11.59	99.12	137.06	152.81	178.16	141.90	7.96	110.63	137.53	147.23	165.21
13	BmrC	3000	20.60	16.50	0.34	8.73	28.96	97.79	28.22	10.59	3.61	20.82	33.48	98.46
18	BmrC	3000	130.67	5.96	113.18	126.27	134.68	151.58	133.37	5.32	115.82	129.56	137.01	149.57
38	BmrC	3000	28.98	3.75	18.70	26.49	31.34	43.29	29.41	5.98	16.12	25.93	31.21	63.40
160	BmrC	3000	140.19	6.38	110.16	136.34	144.66	159.53	144.70	5.32	123.06	141.56	148.28	162.41
182	BmrC	3000	118.61	5.48	101.13	114.80	122.36	139.29	117.88	4.73	104.71	114.46	120.52	136.20
237	BmrC	3000	124.19	6.97	103.12	119.22	129.32	143.89	124.39	5.59	105.24	120.81	128.43	138.17
270	BmrC	3000	101.09	8.78	79.21	95.03	106.22	140.96	94.46	8.96	68.37	87.99	100.95	119.27
2	BmrD	3000	116.04	38.52	2.87	90.20	147.05	179.17	135.89	4.75	124.86	132.37	138.74	156.75
5	BmrD	3000	134.25	7.88	103.09	129.06	139.92	154.73	136.09	8.03	92.76	130.97	141.84	155.22
9	BmrD	3000	143.89	10.03	112.10	136.65	151.44	171.70	145.70	8.95	114.06	139.60	151.79	174.33
15	BmrD	3000	77.08	3.36	64.52	74.81	79.33	89.42	80.24	3.40	68.47	78.08	82.52	91.38
16	BmrD	3000	121.40	5.47	104.16	117.43	125.30	138.71	119.68	4.67	102.34	116.57	122.72	137.63
40	BmrD	3000	42.00	5.71	22.25	38.12	45.84	65.51	40.54	5.98	20.27	36.50	44.78	59.26
41	BmrD	3000	89.11	7.01	70.09	83.86	93.99	112.76	92.26	6.90	73.72	86.86	97.16	115.24
147	BmrD	3000	95.95	7.18	66.43	91.62	100.73	118.39	82.14	6.83	54.72	77.57	86.78	105.57
180	BmrD	3000	82.33	8.34	51.90	76.89	88.63	105.25	81.92	5.81	52.63	79.81	85.55	96.38
251	BmrD	3000	113.96	5.25	94.90	110.51	117.66	129.04	117.94	3.72	101.31	115.75	120.41	129.37
271	BmrD	3000	53.07	6.80	29.21	48.06	58.45	68.22	43.84	4.33	26.23	40.95	46.77	58.77
272	BmrD	3000	46.75	5.89	27.58	42.98	51.07	61.33	44.71	4.03	29.29	42.19	47.37	57.74
328	BmrD	3000	78.14	25.74	14.67	59.08	94.61	168.32	73.12	13.81	35.34	64.53	79.64	138.40
400	BmrD	3000	43.42	25.85	1.85	26.80	50.37	144.69	20.98	12.05	0.25	12.48	26.91	78.24

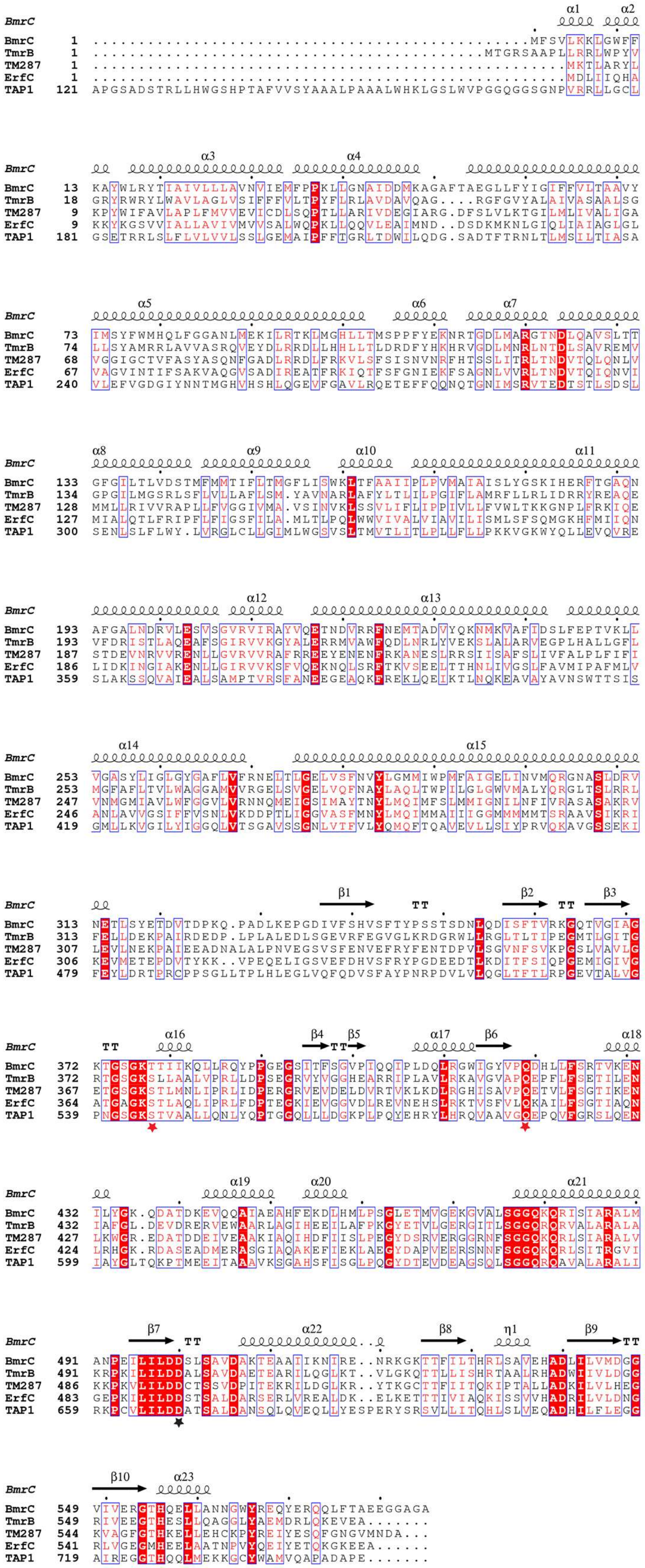
BmrC
TmrB
TM287
ErfC
TAP1

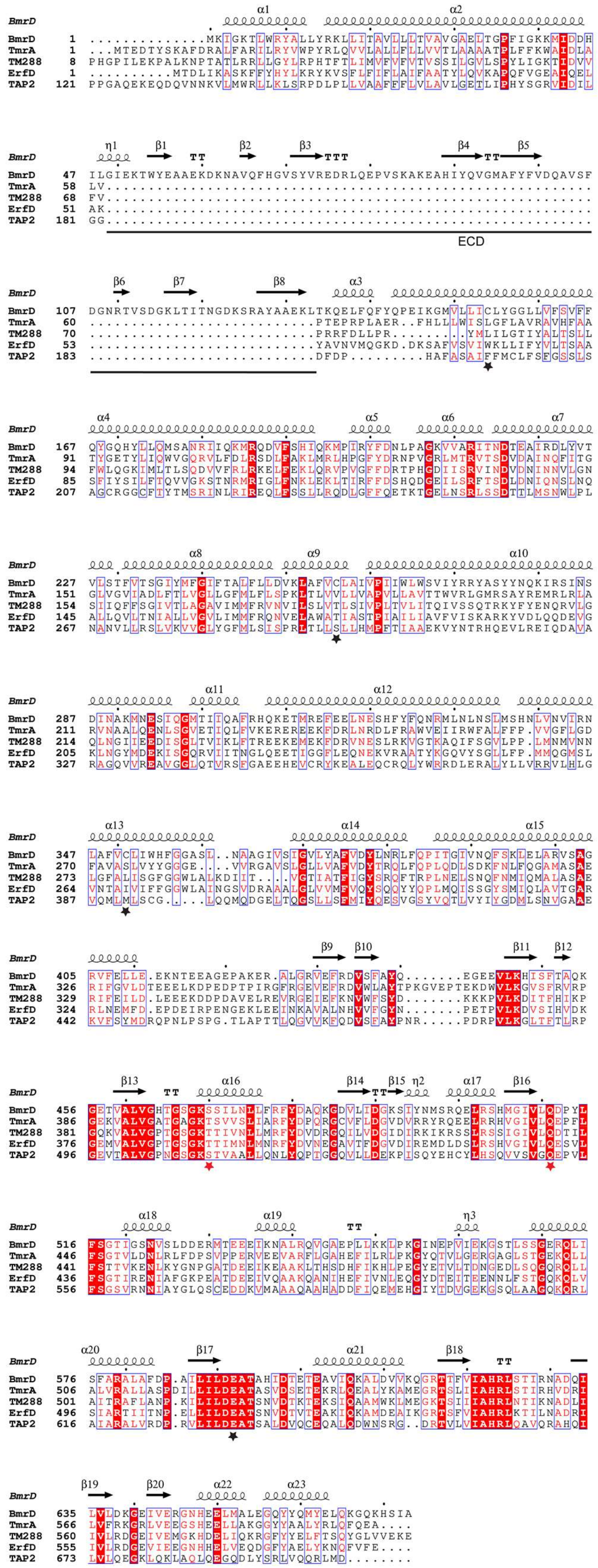
BmrD
TmrA
TM288
ErfD
TAP2
ECD

Supplementary Fig. 1: Sequence alignments of representative heterodimeric ABC transporters. Sequences used for alignment are as follows: BmrC (WP_110109606.1, https://www.ncbi.nlm.nih.gov/protein/WP_110109606.1), BmrD (WP_003233292.1, https://www.ncbi.nlm.nih.gov/protein/WP_003233292.1), TmrA (WP_011173397.1, https://www.ncbi.nlm.nih.gov/protein/WP_011173397.1), TmrB (WP_011173398.1, https://www.ncbi.nlm.nih.gov/protein/WP_011173398.1), TM287 (WP_004083000.1, https://www.ncbi.nlm.nih.gov/protein/WP_004083000.1), TM288 (WP_004083002.1, https://www.ncbi.nlm.nih.gov/protein/WP_004083002.1), ErfC (WP_267561165.1, https://www.ncbi.nlm.nih.gov/protein/WP_267561165.1), ErfD (WP_284955021.1, https://www.ncbi.nlm.nih.gov/protein/WP_284955021.1), TAP1 (NP_000584.3, https://www.ncbi.nlm.nih.gov/protein/NP_000584.3), TAP2 (NP_000535.3, https://www.ncbi.nlm.nih.gov/protein/NP_000535.3). The secondary structural elements of BmrCD are shown on top of the alignment. The strictly conserved residues are highlighted in shaded red boxes and the conserved residues in open red boxes. The conserved, Mg^{2+} coordinating residues are denoted with red stars. The mutations to generate cysteine-less (C154A, C256A, C351A of BmrD) and glutamine substitutions at D500 of BmrC and E592 of BmrD are denoted with black stars. The ECD region is indicated. Sequence alignments were performed by CLUSTALW and the figure was prepared with ESPript3[2].

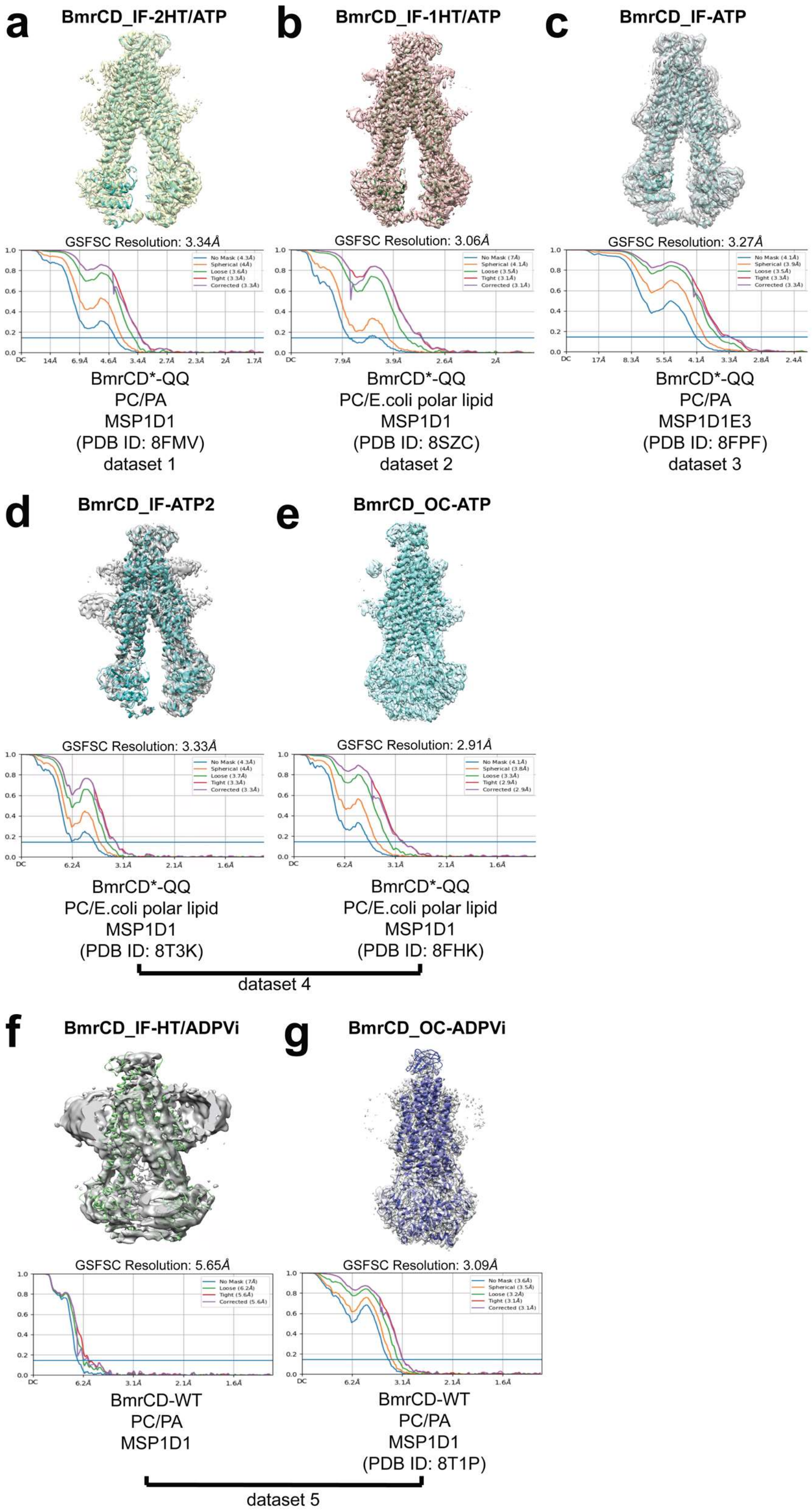

Supplementary Fig. 2: Conformational gallery of BmrCD.

Seven cryo-EM maps from 5 datasets are shown, and the cartoon models are fitted into the maps (For more information see Methods). Original maps estimated resolution is based on Fourier shell correlation (FSC) curves, **a**-**e** are original maps after local refinement in cryoSPARC; **f** and **g** are original maps after NU refinement in cryoSPARC. The BmrCD constructs, lipid composition of the nanodiscs and MSP used for reconstitution are shown below the maps. BmrCD*-QQ and BmrCD-WT refer to cysteine-less BmrCD in QQ background and wild-type BmrCD respectively.

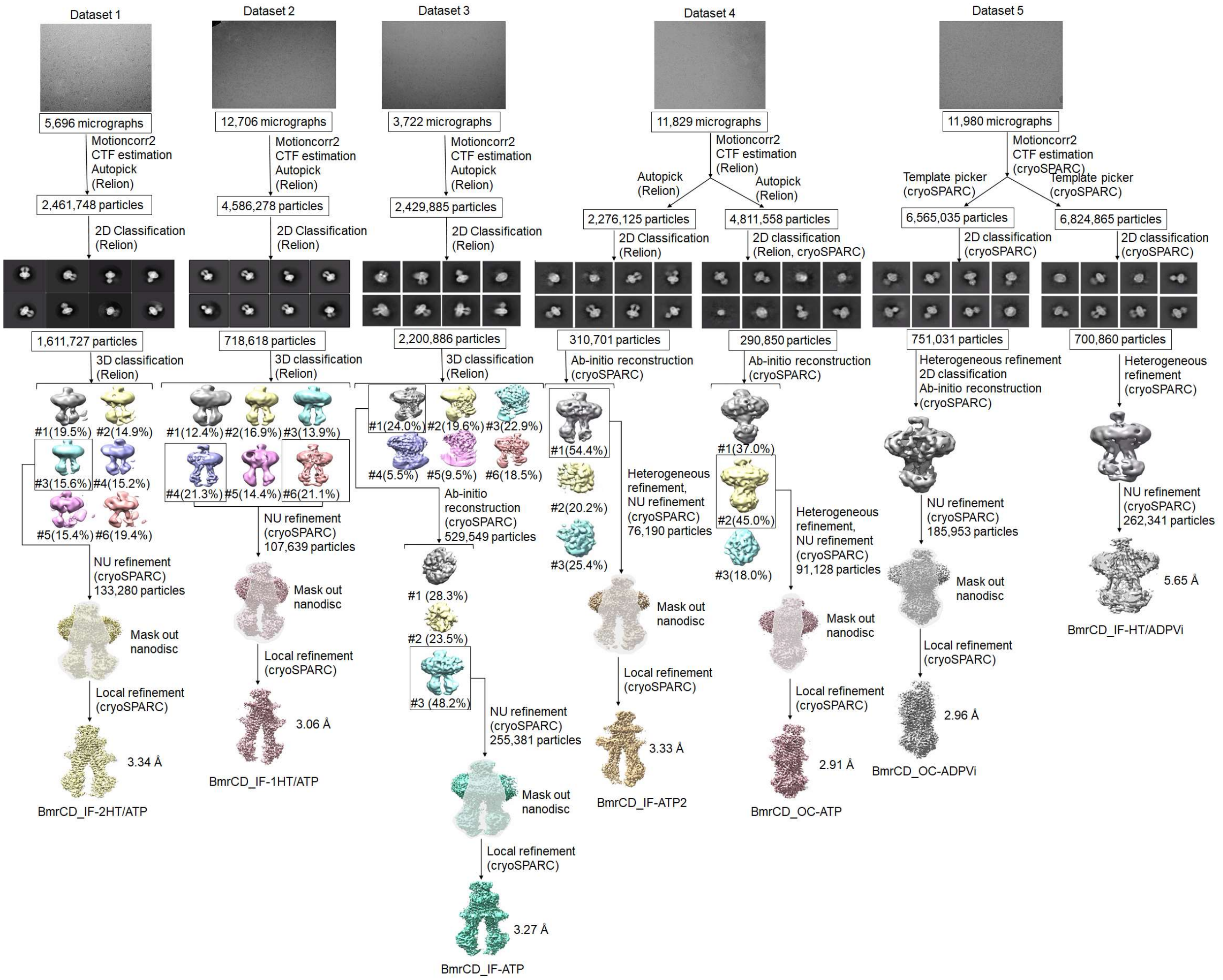

Supplementary Fig. 3: Cryo-EM data processing workflow for BmrCD datasets. The diagram schematically depicts all the five BmrCD cryo-EM datasets that were collected in this study and the computational processing steps used to reconstruct the seven BmrCD original maps: BmrCD_IF-2HT/ATP, BmrCD_IF-1HT/ATP, BmrCD_IF-ATP, BmrCD_IF-ATP2, BmrCD_OC-ATP, BmrCD_OC-ADPVi, and BmrCD_IF-HT/ADPVi. Programs used for each step are noted, and the representative micrographs, 2D and 3D images are shown above.

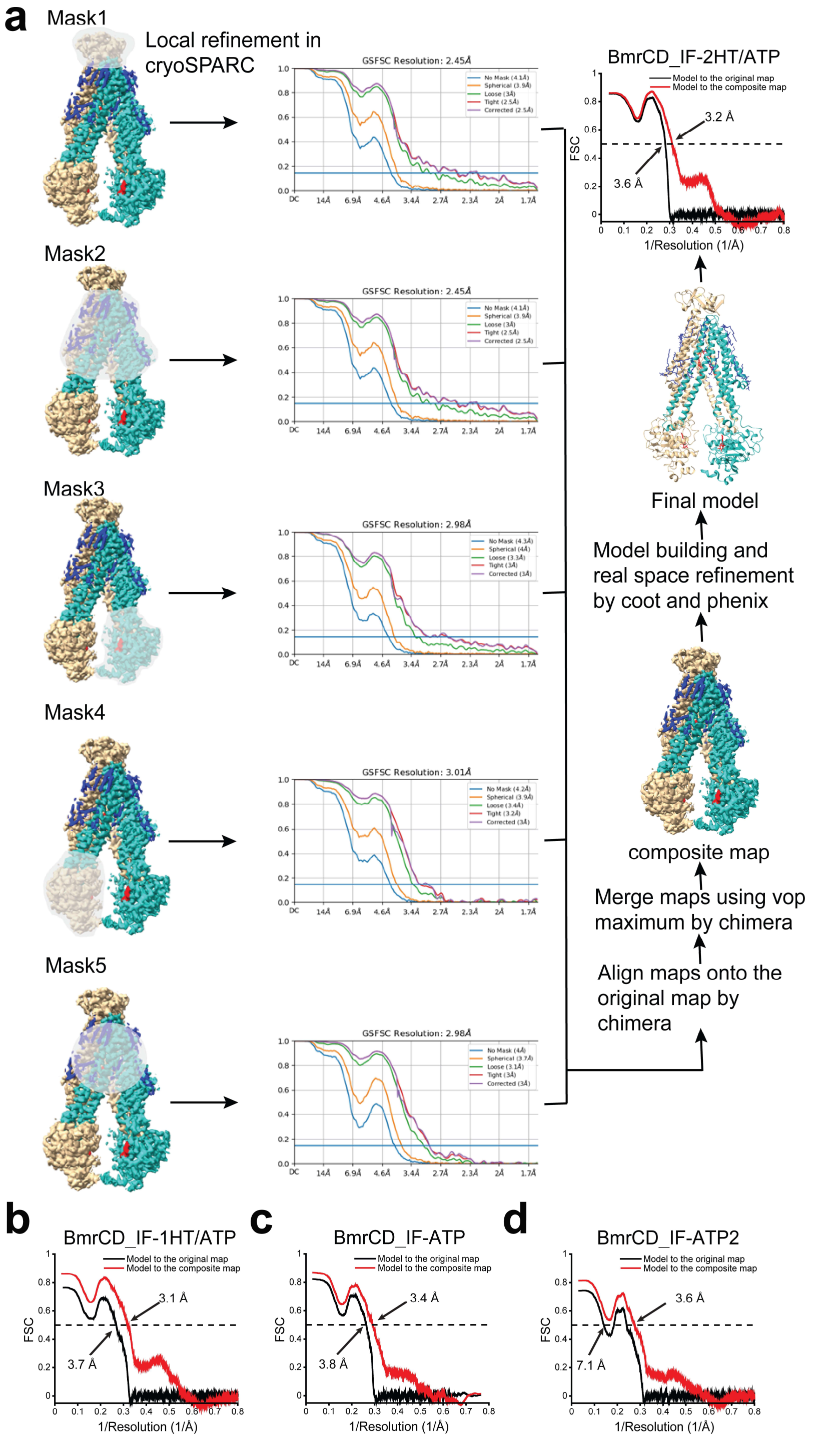

Supplementary Fig. 4: Diagram showing the local refinement strategy for BmrCD inward facing conformations.

a Diagram showing the local refinement strategy for BmrCD_IF-2HT/ATP. Masks are shown transparent, and subunits colored as in Fig. 1. Fourier shell correlation (FSC) curves for each mask after local refinement in cryoSPARC are shown. FSC curves of

the refined model versus the original map after NU refinement in cryoSPARC (black) and the composite map (red) are shown at the final step in the diagram. **b**, **c**, and **d** Similar local refinement strategy was applied and the FSC curves of the refined model versus the original map (black) and the composite map (red) are plotted for BmrCD_IF-1HT/ATP (**b**), BmrCD_IF-ATP (**c**), BmrCD_IF-ATP2 (**d**).

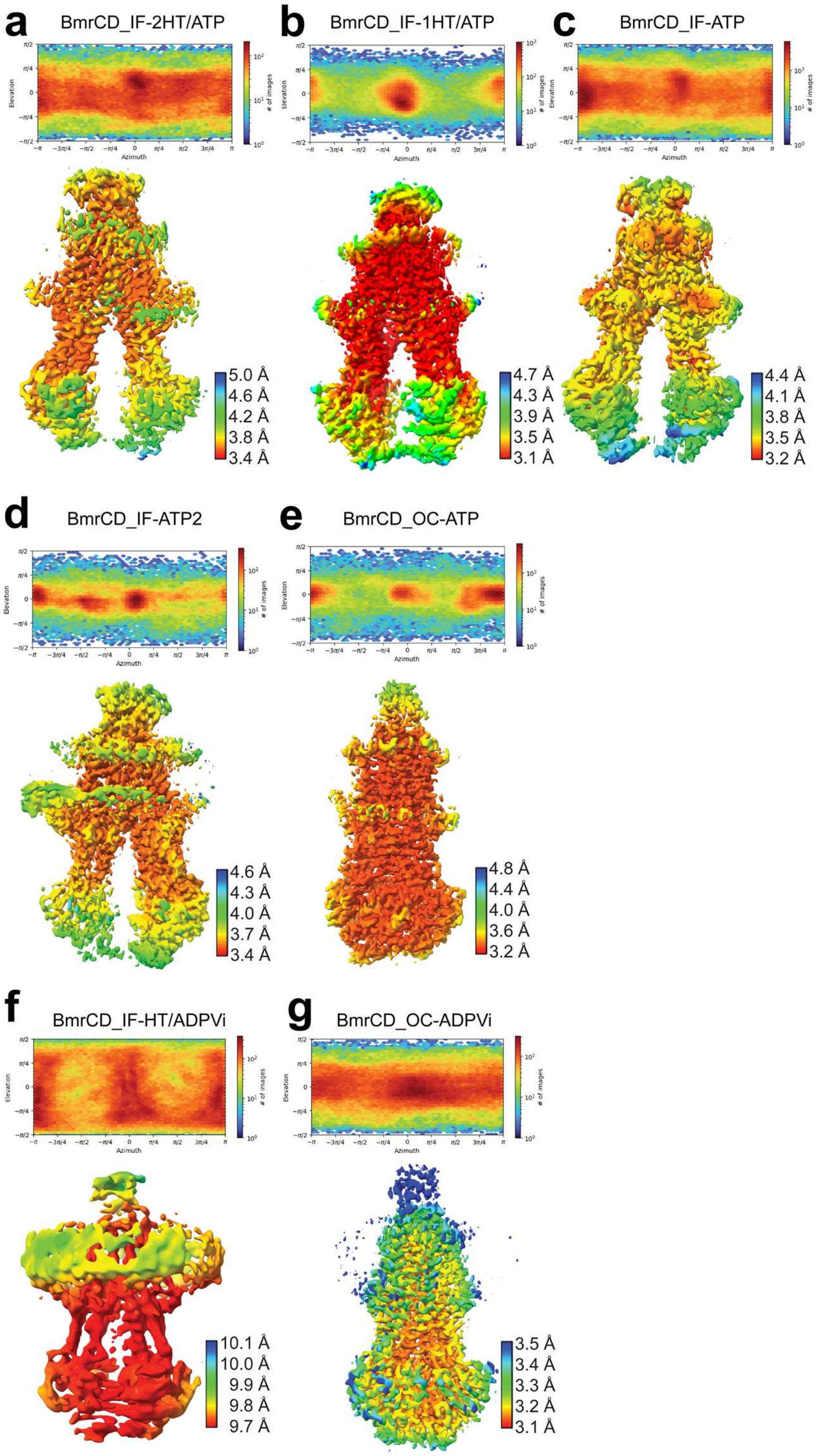

Supplementary Fig. 5: Angular distribution of particles and local resolution maps for BmrCD.

a-**e**, and **g** angular distribution of particles (upper panel) and local resolution map (lower panel) for original maps after local refinement in cryoSPARC for BmrCD_IF-2HT/ATP (**a**),

BmrCD_IF-1HT/ATP (**b**), BmrCD_IF-ATP (**c**), BmrCD_IF-ATP2 (**d**), BmrCD_OC-ATP (**e**), BmrCD_OC-ADPVi (**g**). **f** angular distribution of particles (upper panel) and local resolution map (lower panel) for original maps after NU refinement in cryoSPARC for BmrCD_IF-HT/ADPVi.

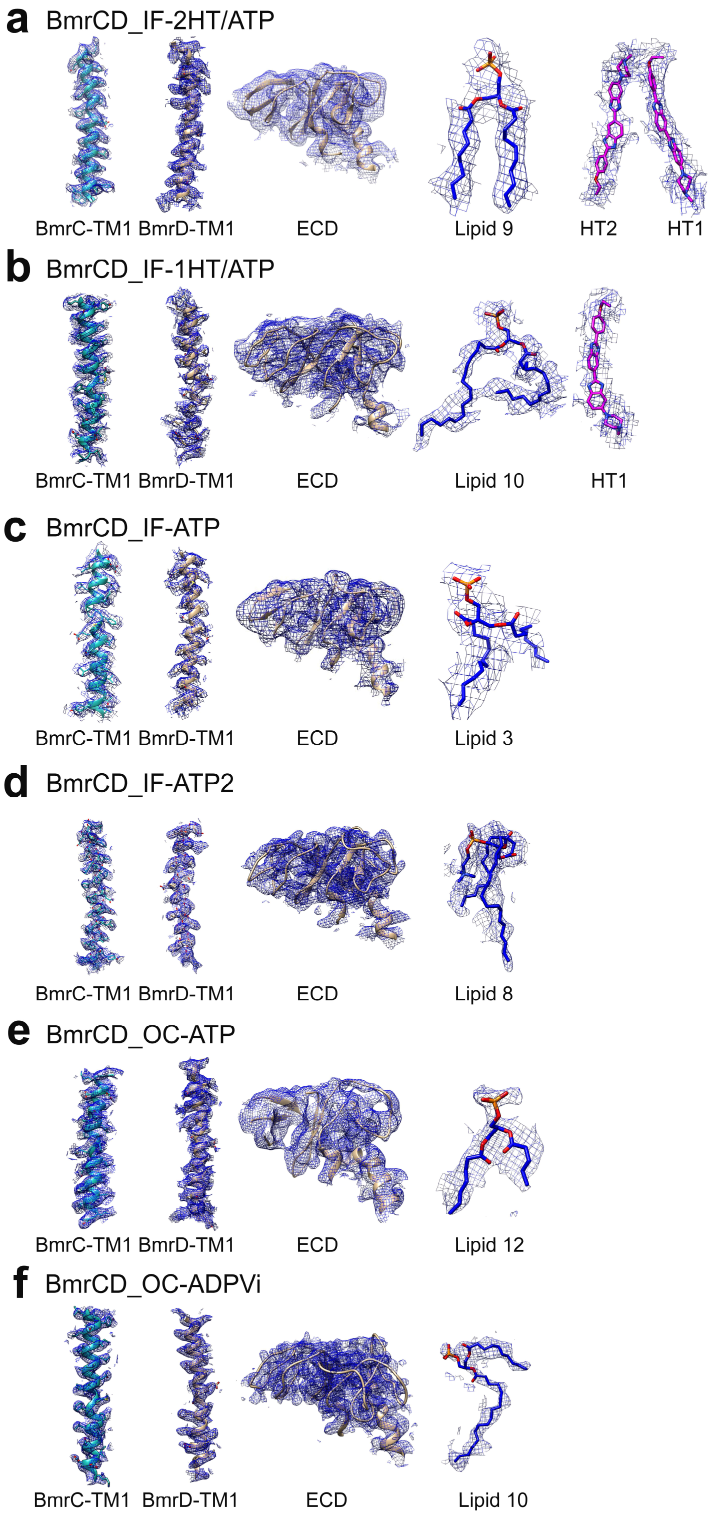

Supplementary Fig. 6: Representative cryo-EM densities for BmrCD.

Representative cryo-EM densities for BmrCD structure models: BmrCD_IF-2HT/ATP (**a**), BmrCD_IF-1HT/ATP (**b**), BmrCD_IF-ATP (**c**), BmrCD_IF-ATP2 (**d**), BmrCD_OC-ATP (**e**),

BmrCD_OC-ADPVi (**f**). Transmembrane domain helices TM1 of BmrC and BmrD are selected for illustration of the strong side-chain densities. Extracellular domains (ECD), selected lipids molecules (labeled with their ID in the structures) and substrates Hoechsts (HT-1 and HT-2) are shown along with their respective densities.

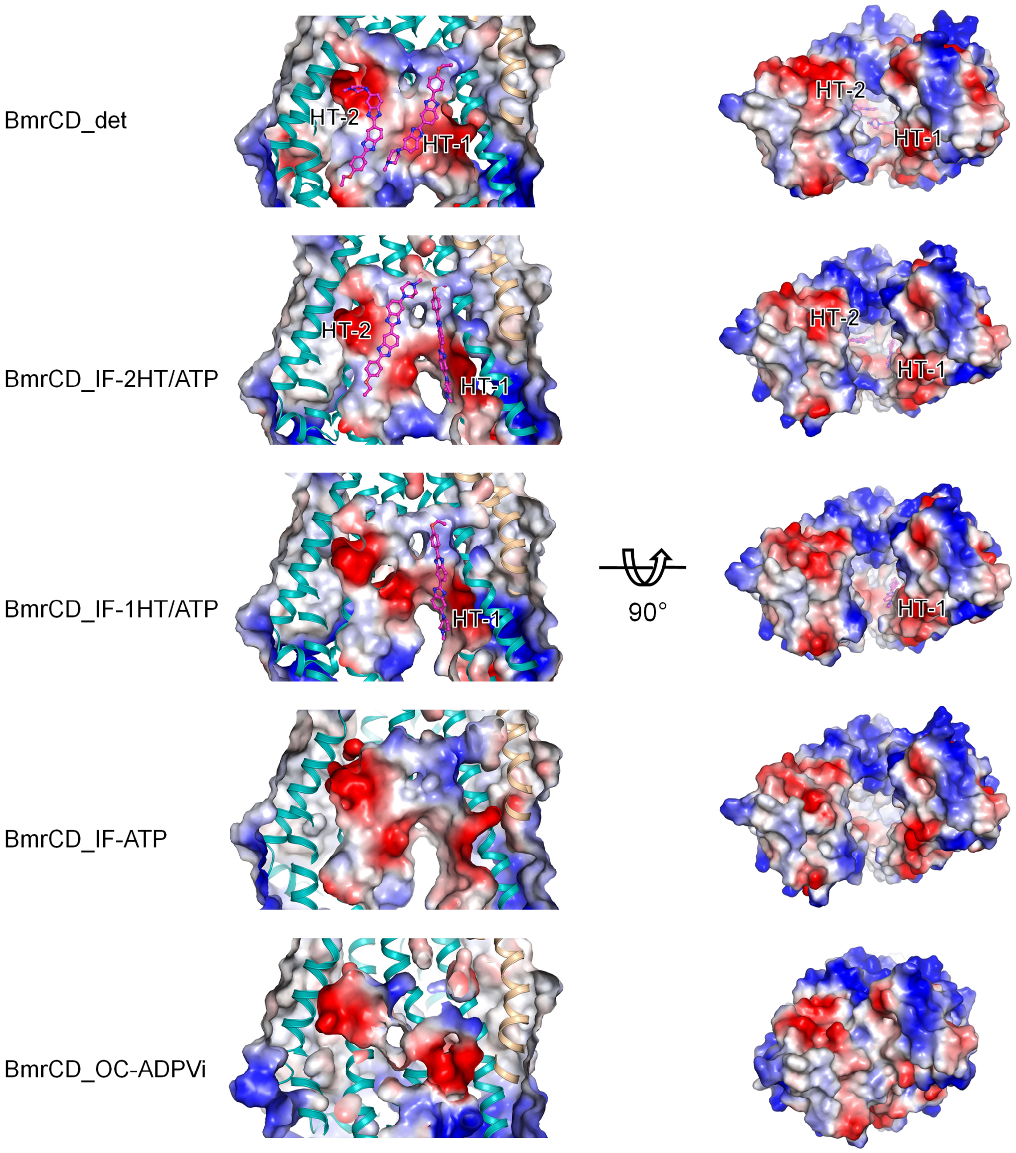

Supplementary Fig. 7: Electrostatics analysis of Hoechst-binding pocket.

Upper panels show side views of the electrostatic surface of the substrate Hoechst-binding pocket of BmrCD_det (PDB ID-7m33, https://www.rcsb.org/structure/7M33), BmrCD_IF-2HT/ATP, BmrCD_IF-1HT/ATP, BmrCD_IF-ATP, and BmrCD_OC-ATP. The lower panels show the bottom view of the TMD. The surfaces are colored from blue (positively charged regions) to red (negatively charged regions). Neutral regions are shown as white. BmrCD is shown in cartoon and Hoechst is shown in stick. Two Hoechst molecules are labeled as HT-1 and HT-2.

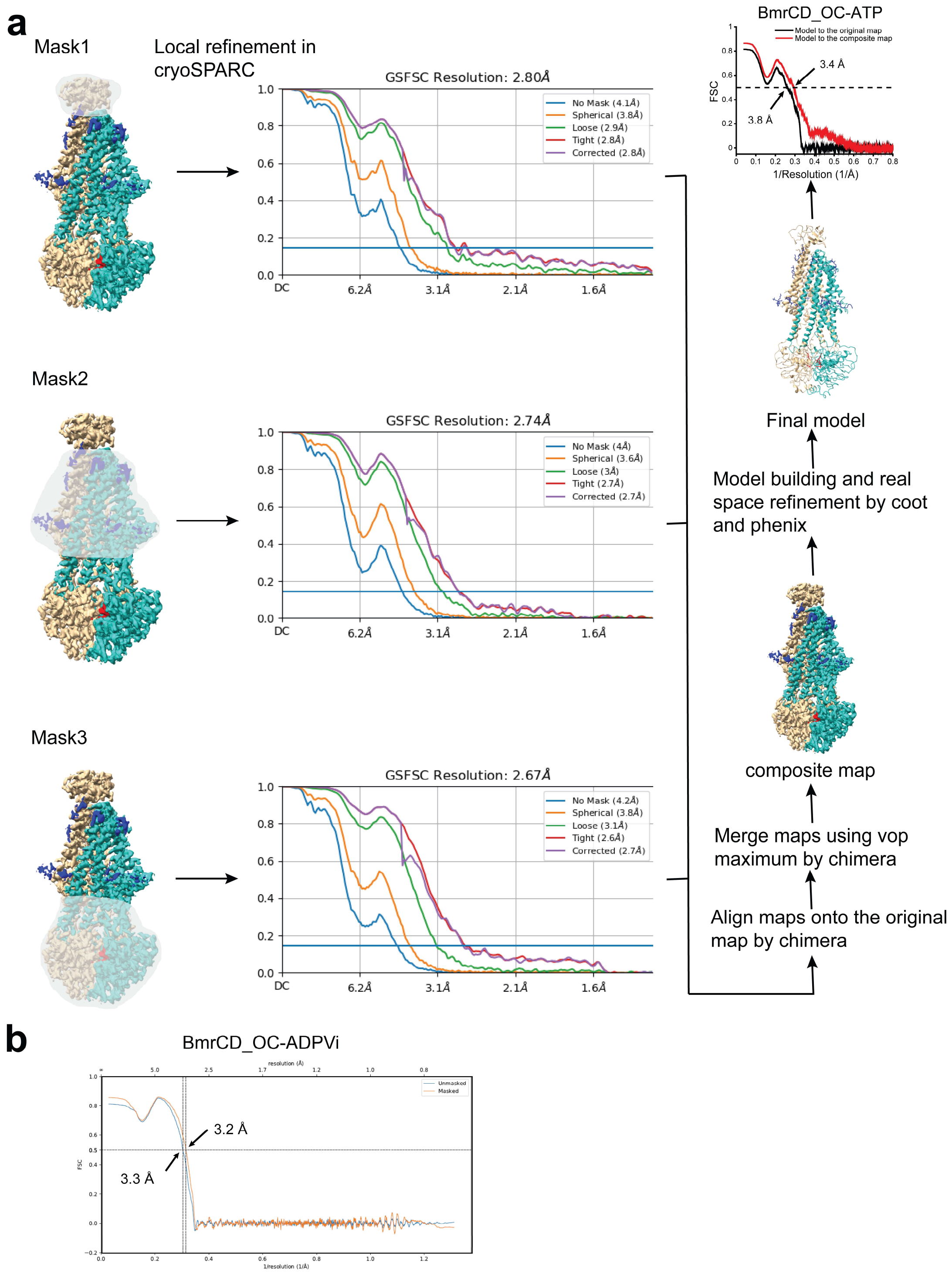

Supplementary Fig. 8: Refinement strategies for BmrCD_OC-ATP and BmrCD_OC-ADPVi.

a Local refinement strategy for BmrCD_OC-ATP. Masks are shown transparent, and subunits colored as in Fig. 1. Fourier shell correlation (FSC) curves for each mask after local refinement in cryoSPARC are shown. FSC curves of the refined model versus the original map after NU refinement in cryoSPARC (black) and the composite map (red) are shown at the final step in the diagram. **b** Real-space refinement for BmrCD_OC-ADPVi. Fourier shell correlation curves show model fit after Real-space refinement for BmrCD_OC-ADPVi. The local refinement strategy to obtain a composite map was not applied for BmrCD_OC-ADPVi (See Supplementary Table 1 and Supplementary Fig. 2).

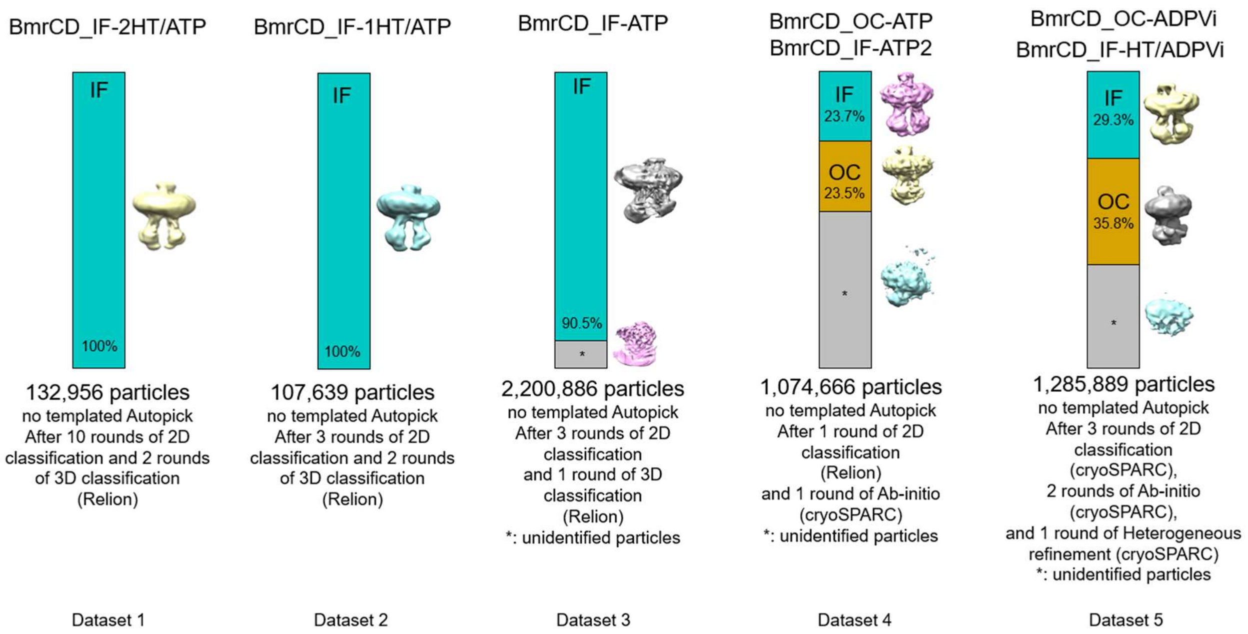

Supplementary Fig. 9: Particle distributions in the 5 datasets.
The percentage of the particles' distributions in each dataset are listed, and the representative images of the conformations are shown.

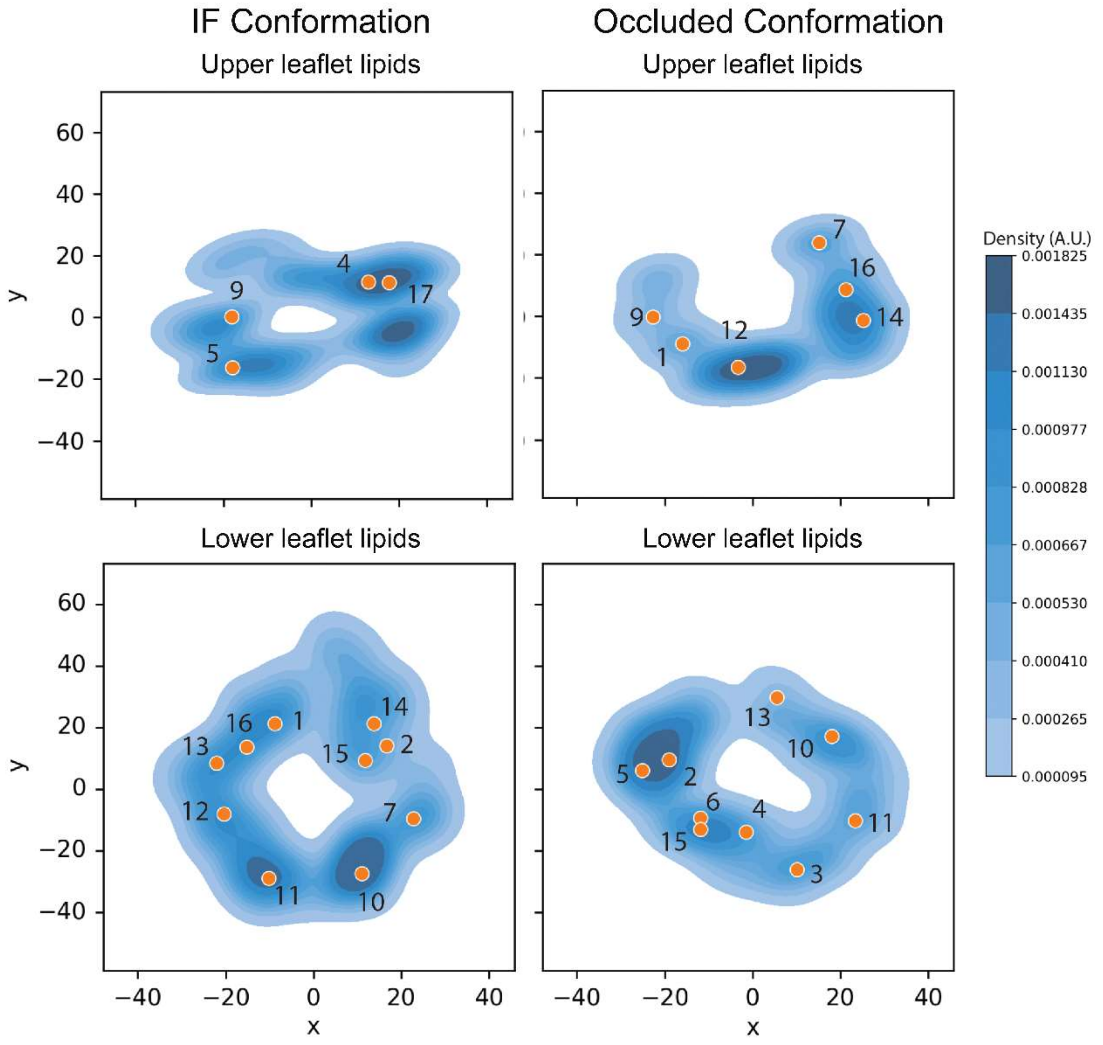

Supplementary Fig. 10: Lipids distributions in BmrCD cryo-EM structures. Kernel density estimation was used to plot the lipids distribution in BmrCD cryo-EM structures excluding lipids without observable headgroup. The lipid positions of phosphorus atoms from BmrCD_IF-2HT/ATP are marked in orange dots with numbers for reference while those for BmrCD_IF-1HT/ATP, BmrCD_IF-ATP, and BmrCD_IF-ATP) are represented by the lipid density. Similarly, lipid density for the two occluded conformations (BmrCD_OC-ATP and BmrCD_OC-ADPVi) are plotted. The lipid positions of phosphorus atoms from BmrCD_OC-ATP are marked in orange dots and numbers for reference. The regions with oval darker blue densities highlight conserved lipid binding sites (lipids 4, 17, 10, and 11 in IF conformation; 12, 14, 2, and 5 in occluded conformation), and the regions with smeared densities nearby indicate less conserved lipid binding sites in the cryo-EM structures.

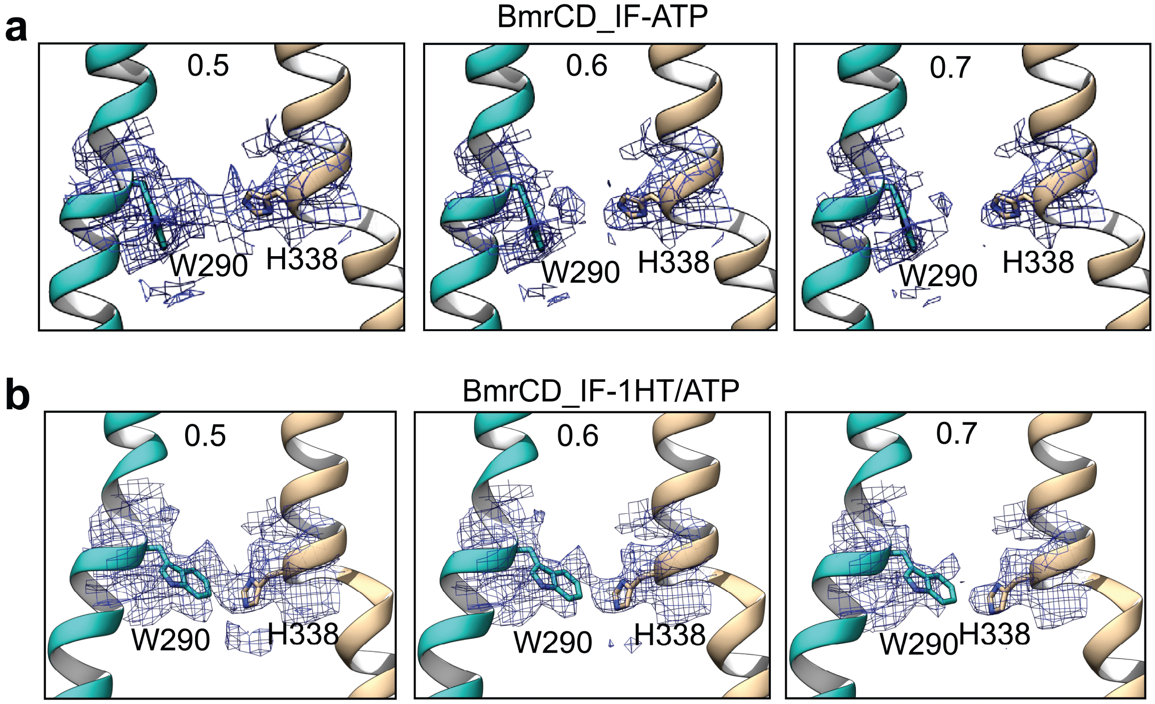

Supplementary Fig. 11: Cryo-EM densities of WH-latch in BmrCD_IF-ATP and BmrCD_IF-1HT/ATP.

The densities are contoured at the three contour levels in Chimera as indicated. The density of W290 in BmrCD_IF-ATP (**a**) indicates that W290 may also adopt a locked latch conformation as in BmrCD_IF-1HT/ATP (**b**) in addition to the major open conformation that blocks HT binding.

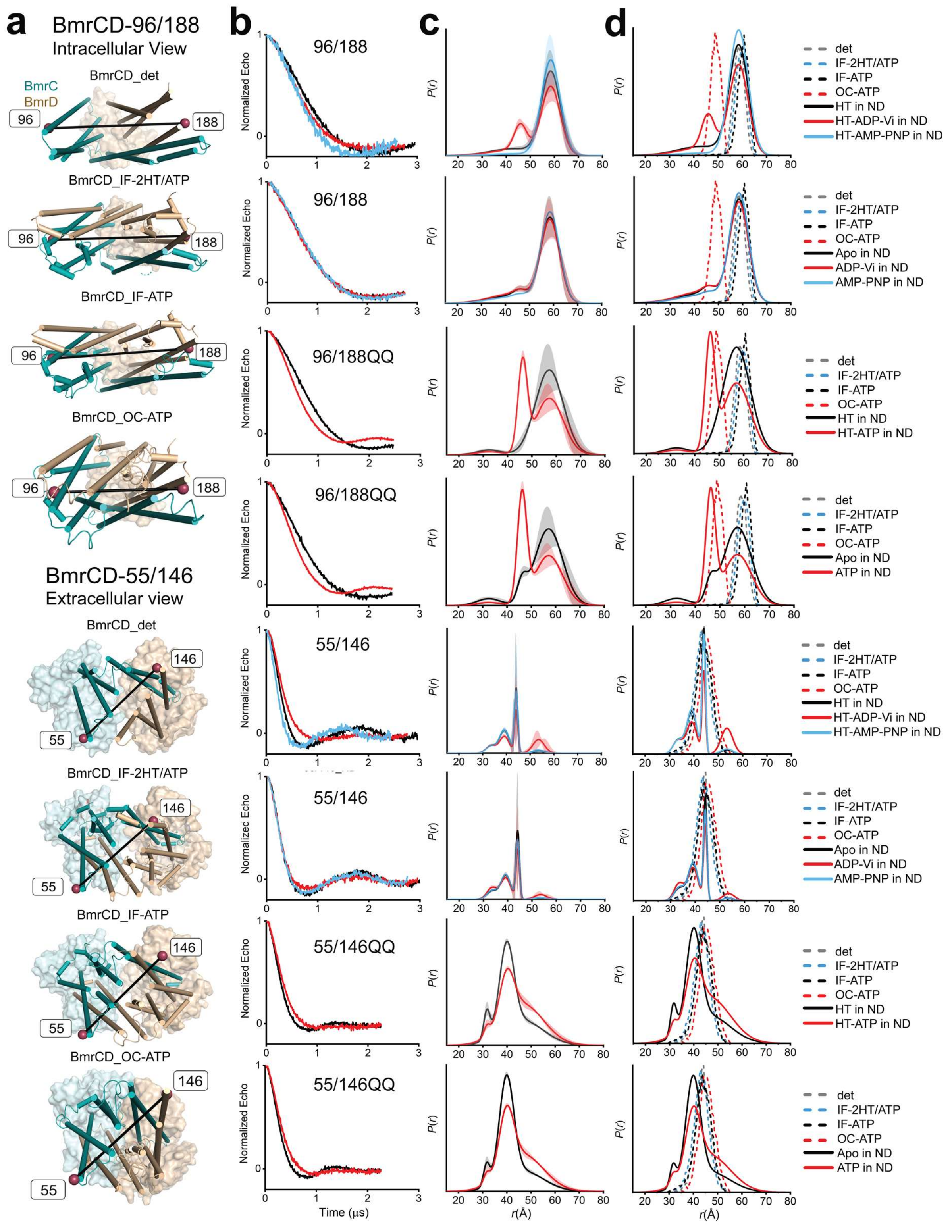

Supplementary Fig. 12: DEER decay signals for spin-labeled BmrCD mutants in the TMD.

a Cartoon representation of BmrCD highlighting the spin-labeled positions.
b Normalized Echo decay intensity curves. **c** Distance distribution analyzed as described in the methods. The light color bands represent confidence bands. **d** Distance distribution from DEER (solid lines) compared with predicted distribution from cryo-EM structures (dashed lines).

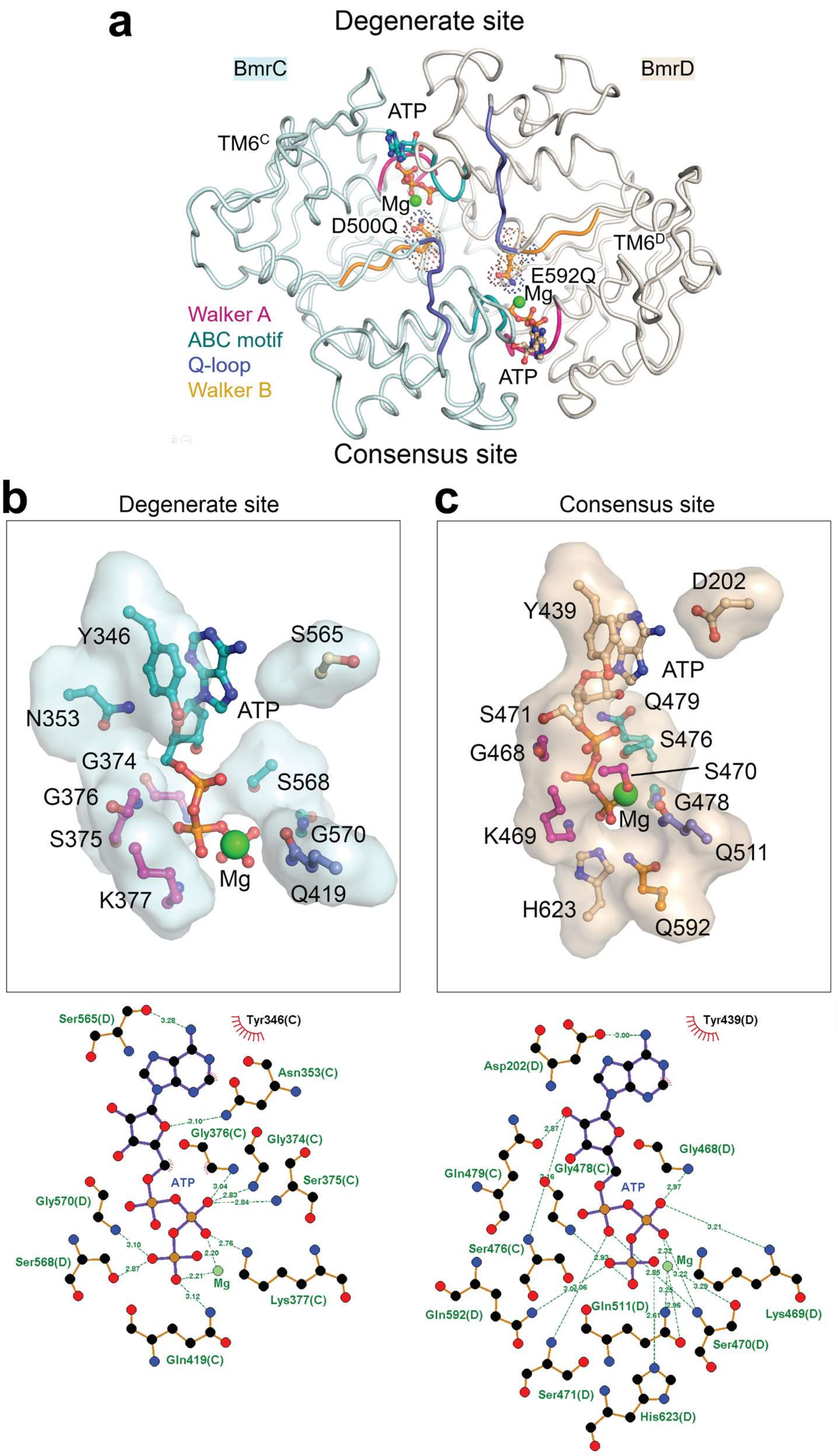

Supplementary Fig. 13: Asymmetric coordination of ATP in the OC conformation (BmrCD_OC-ATP).

a Close-up view of the degenerate and consensus NBSs. BmrCD is shown in ribbon representation with catalytic residues side chains and ATP represented by sticks. Conserved motifs are highlighted. **b** and **c** The atomic features of the complete ATP-binding pockets in the degenerate NBS (**b**), and the consensus NBS (**c**). The key residues from the conserved motifs are shown in sticks in the same colors, and LigPlot schematics of ATP binding are shown below. Interactions between ATP and BmrCD_OC-ATP at the degenerate and consensus NBSs were plotted by LigPlot[1] with default hydrogen-bond calculation parameters setting (Maximum H-A distance is set as 2.70 Å and maximum D-A distance is set as 3.35 Å). Electrostatic and hydrophobic interactions are shown in dashes and eyelashes, respectively.

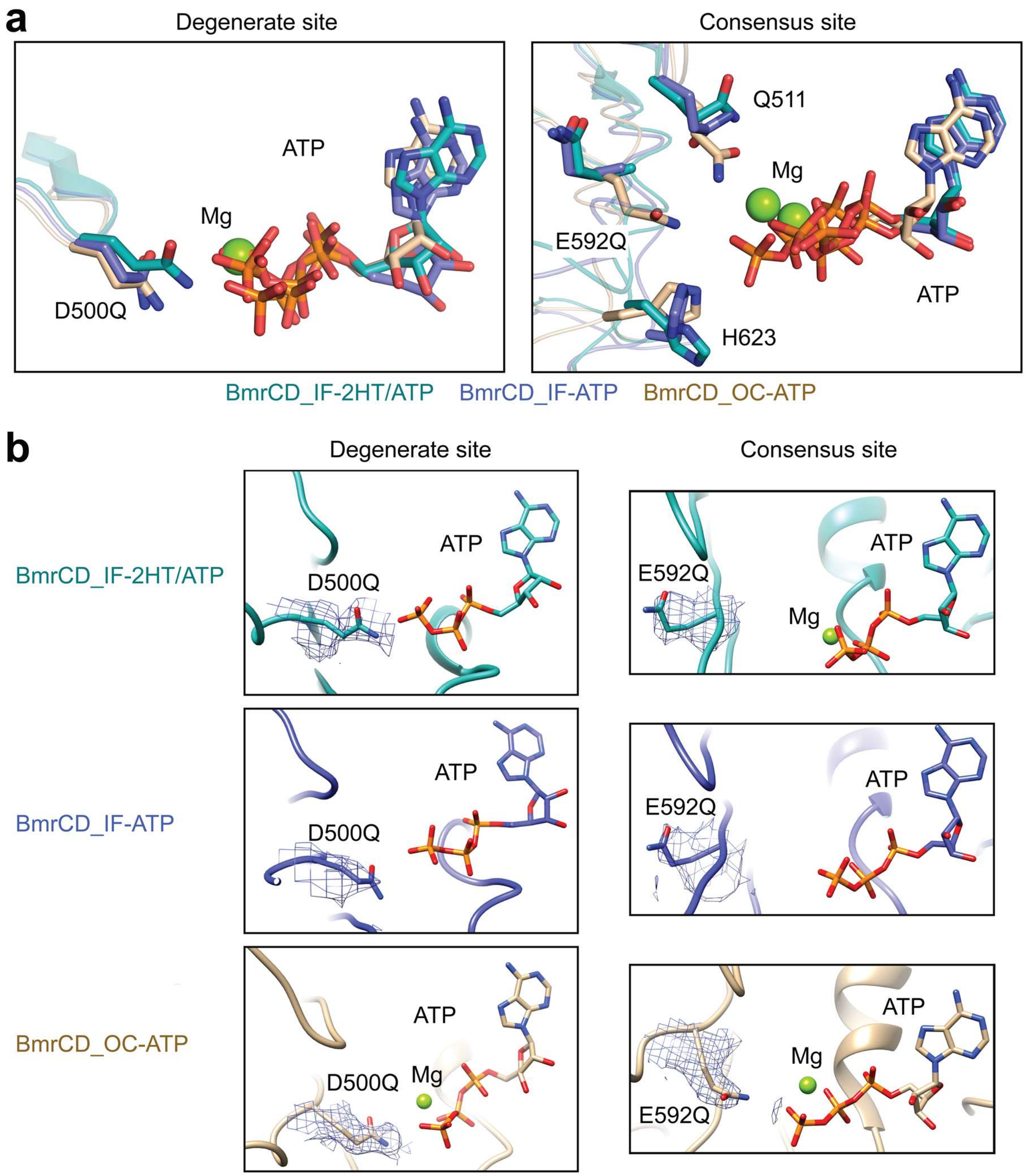

Supplementary Fig. 14: Configurations of the catalytic residues in the NBSs.

a Superimposition of BmrCD NBDs to highlight the catalytic residues D500Q and E592Q in degenerate NBS and consensus NBS, respectively. **b** cryo-EM Densities of the catalytic residues.

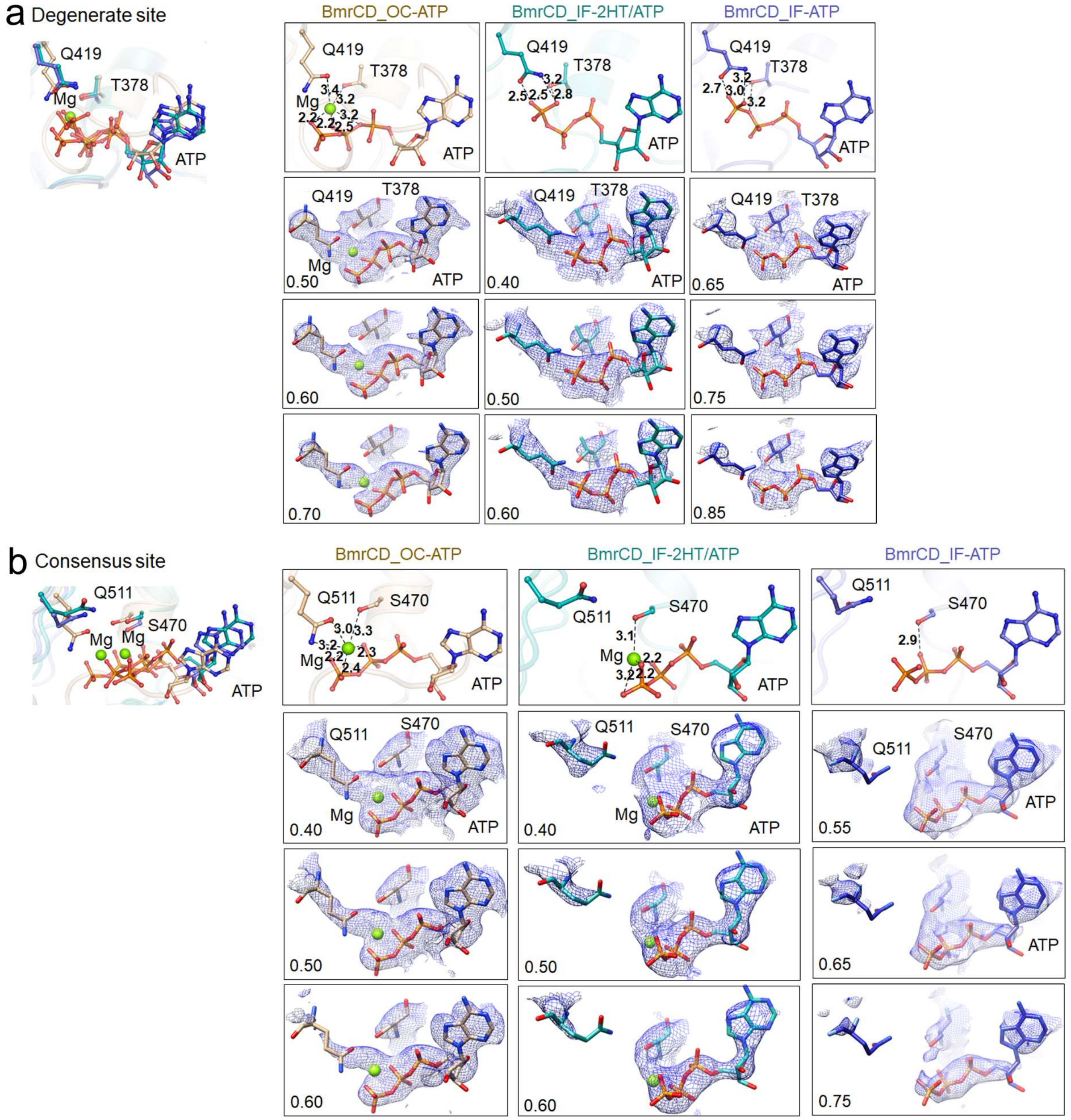

Supplementary Fig. 15: Magnesium ions in BmrCD conformations.

a Coordination of Mg^{2+} in the degenerate NBS. The distance of the Mg^{2+} to its coordinating residues and relative distances (Å) are shown in boxed pictures and cryo-EM densities are shown below at three chimera threshold levels. Superimposition of the coordination sites are shown left. **b** Similar analysis of the coordination of Mg^{2+} in the consensus NBS.

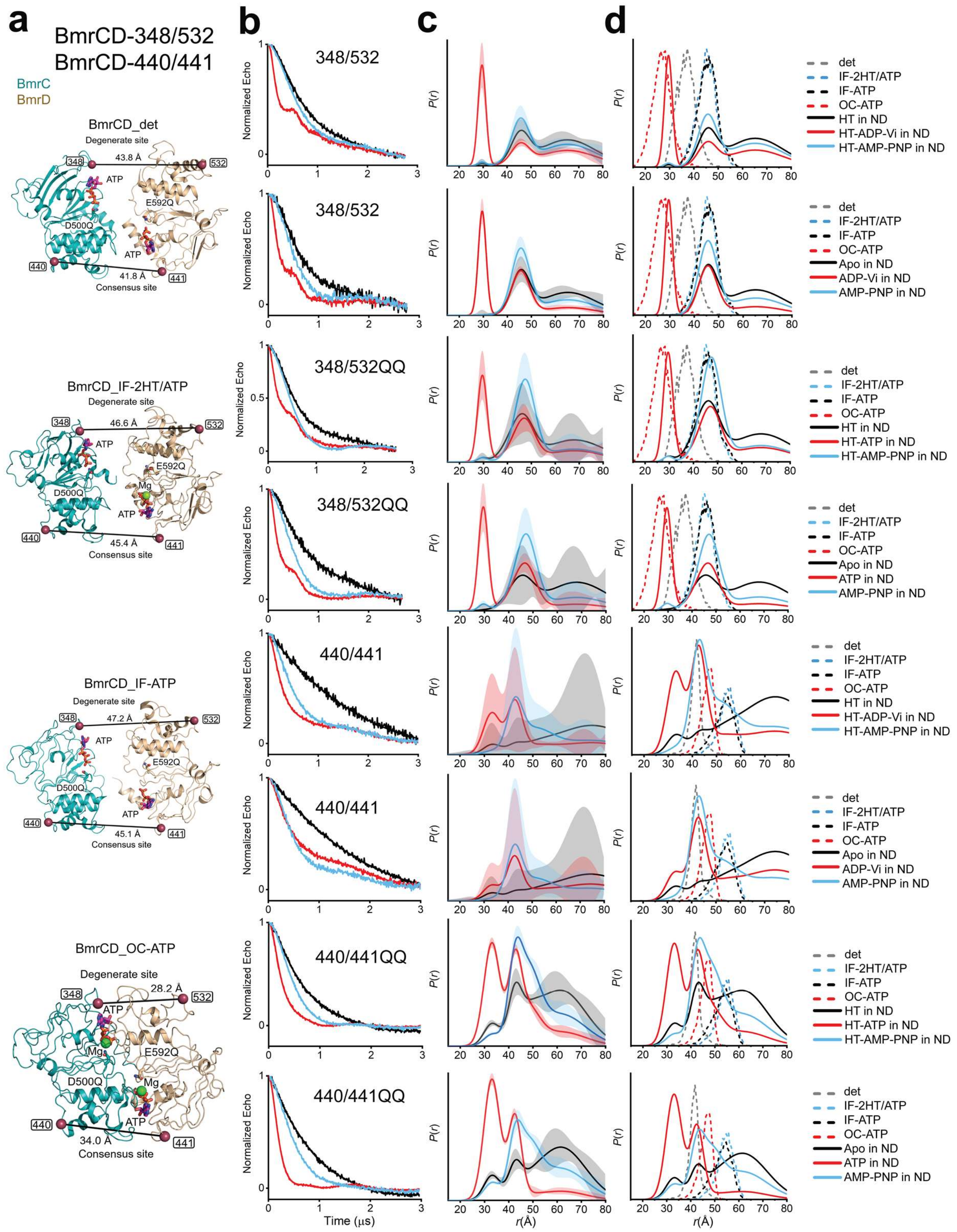

Supplementary Fig. 16: DEER decay signals for spin-labeled BmrCD mutants in the NBD.

a Cartoon representations of BmrCD highlighting the spin-labeled positions.

b Normalized Echo decay intensity curves. **c** Distance distribution analyzed as described in the methods. The light color bands represent confidence bands. **d** Distance distribution from DEER (solid lines) compared with predicted distribution from cryo- EM structures (dashed lines).

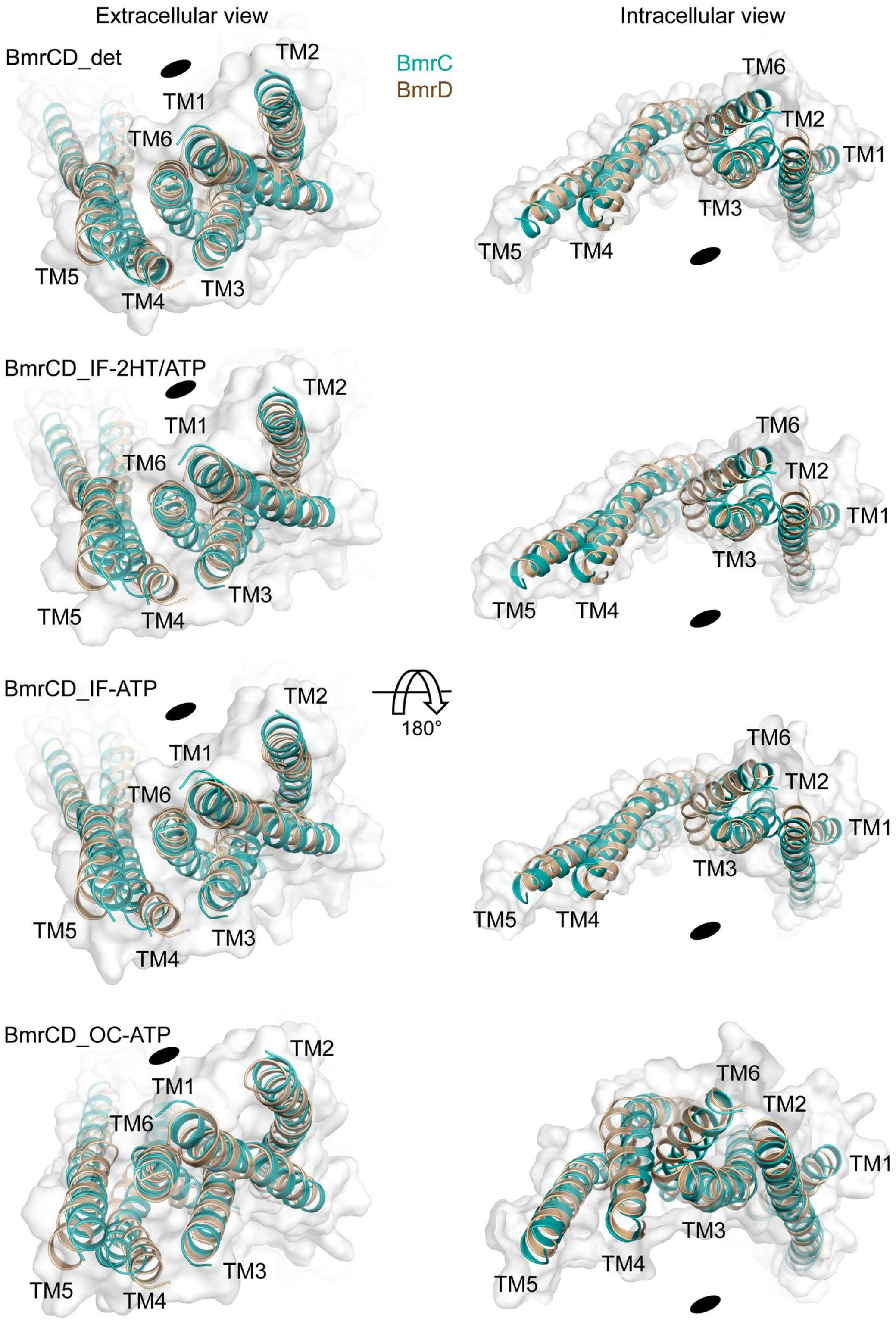

Supplementary Fig. 17: Superimposition of transmembrane helices from BmrC and BmrD highlighting asymmetry.

Transmembrane helices are shown in cartoon and labeled. The black ellipse represents the symmetry axis.

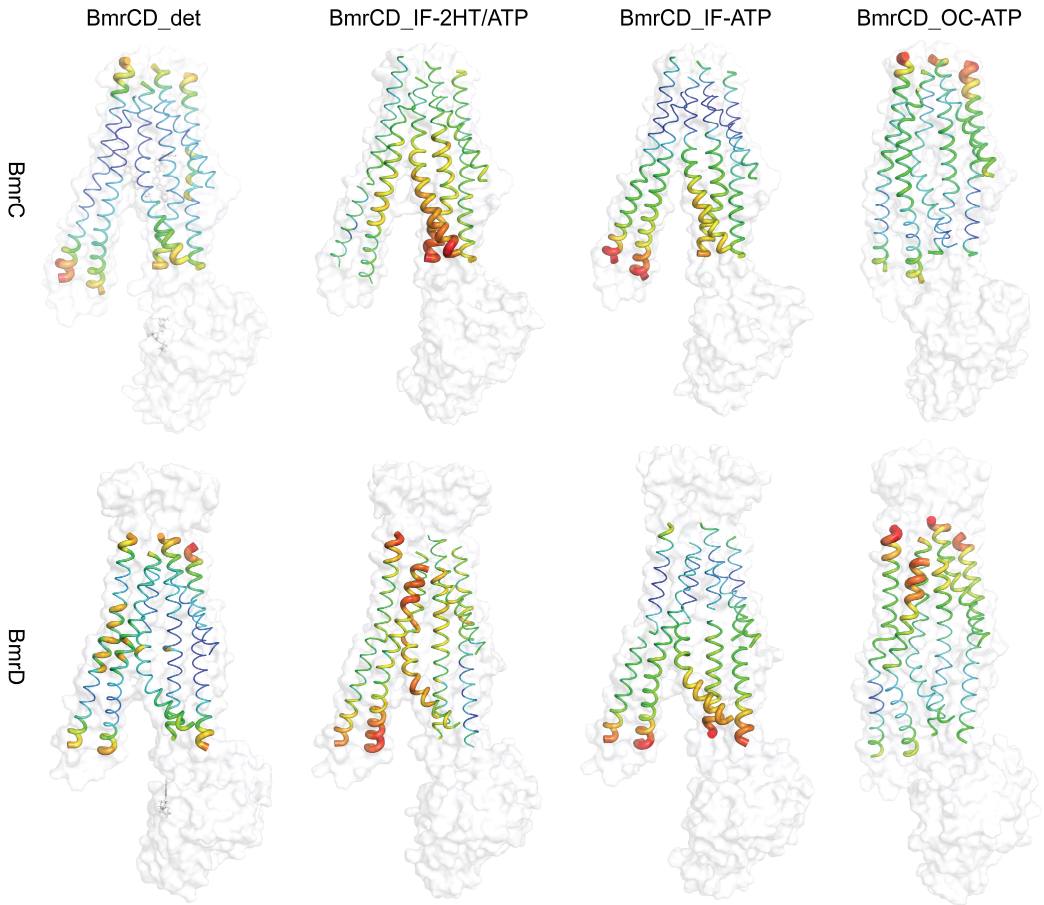

Supplementary Fig. 18: B-factor maps of transmembrane helices.

B-factor maps of the transmembrane helices of BmrC and BmrD are generated by “b factor putty” in PyMol.

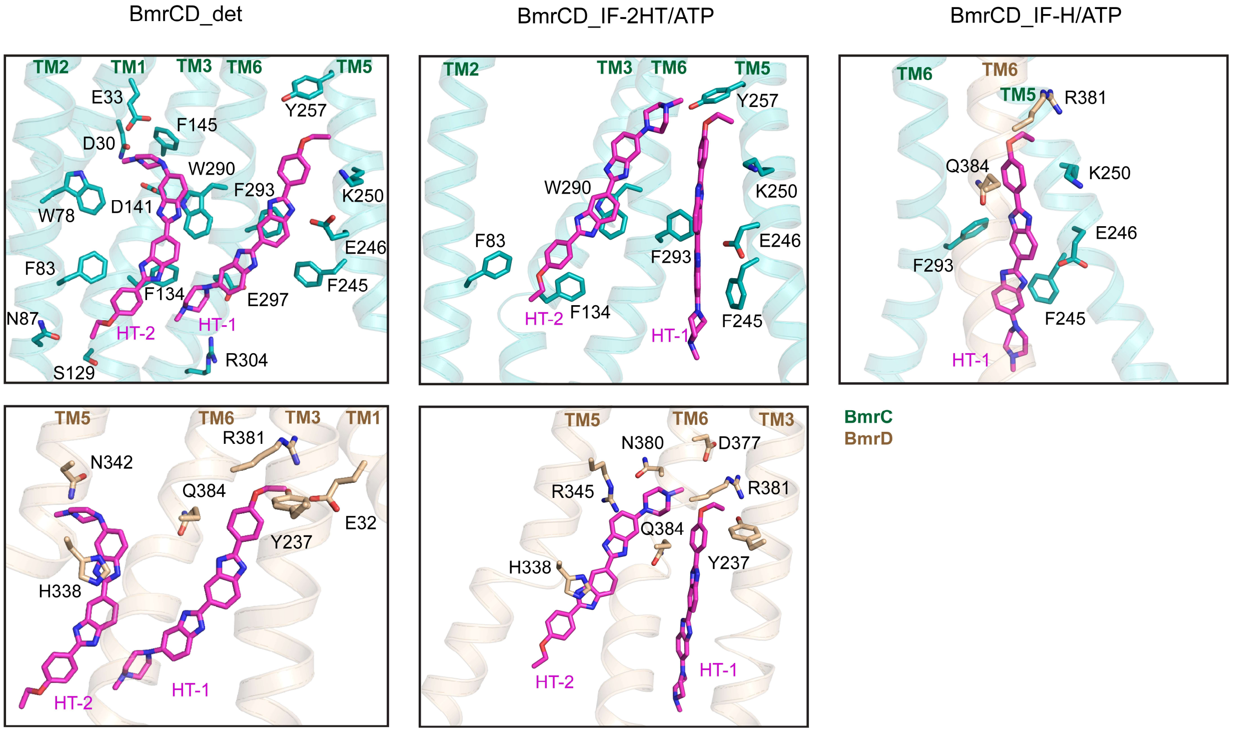

Supplementary Fig. 19: Hoechst-binding pockets in BmrCD_det, BmrCD_IF-2HT/ATP, and BmrCD_IF-1HT/ATP.

Representative residues of the binding pocket of Hoechst (HT) are shown as green/tan sticks. Substrate Hoechsts (HTs) are shown as magenta sticks. Upper panels and lower panels in BmrCD_det and BmrCD_IF-2HT/ATP are in zoomed side view of the interactions with BmrC and BmrD, respectively.

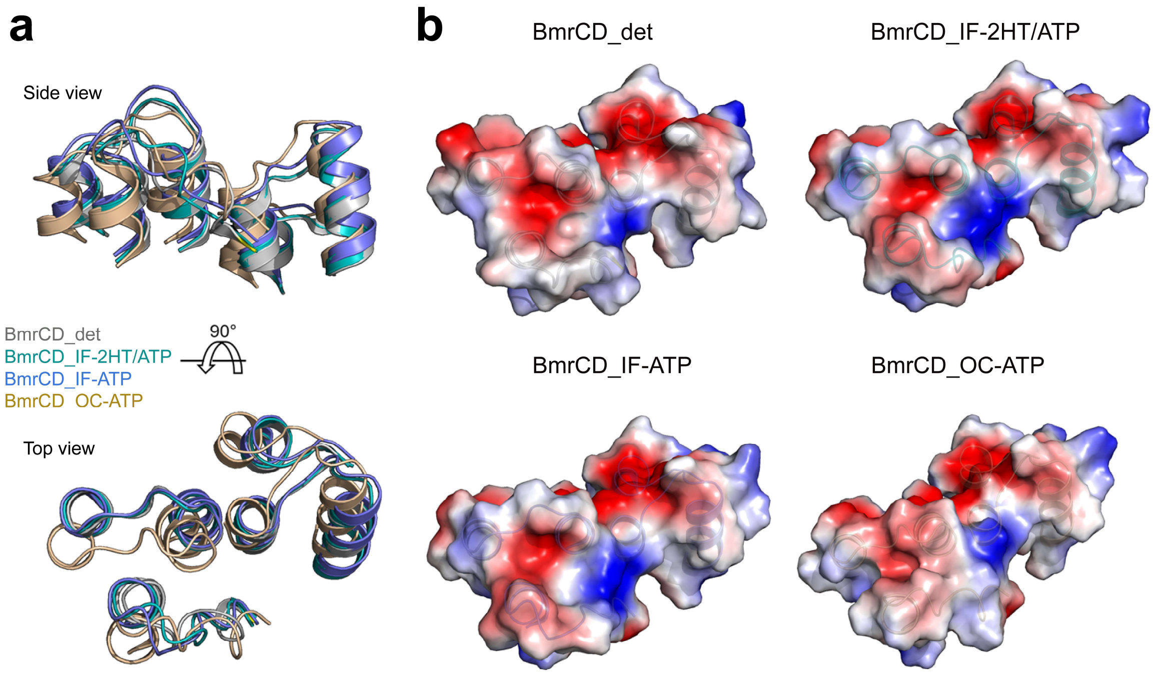

Supplementary Fig. 20: The extracellular gate of BmrCD.

a Superimposition of the extracellular gate in cartoon. **b** Electrostatics surface analysis of the extracellular gate of BmrCD.

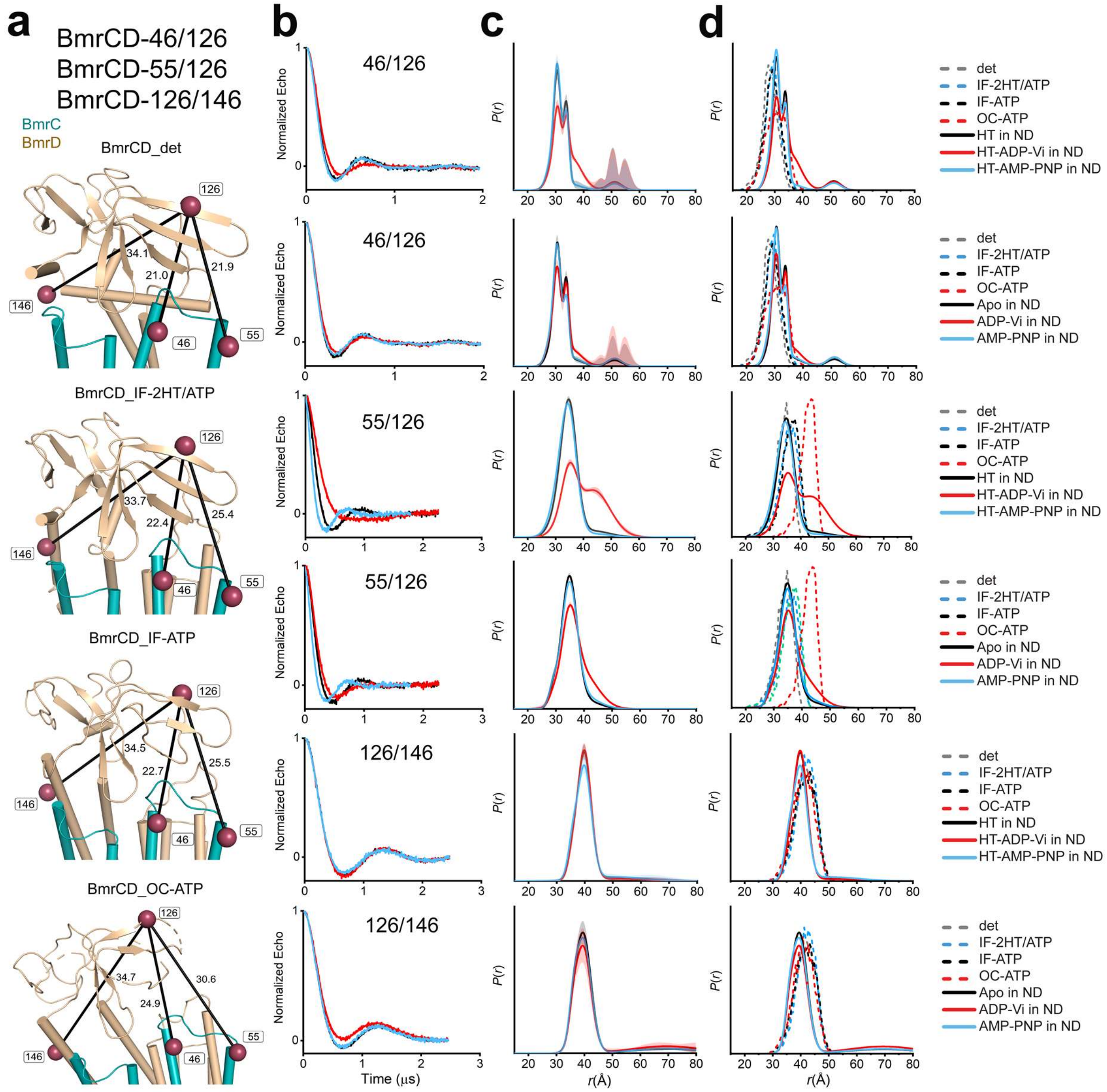

Supplementary Fig. 21: DEER decay signals for spin-labeled BmrCD mutants in the ECD.

a Cartoon representation of BmrCD highlighting the spin-labeled positions.
b Normalized Echo decay intensity curves. **c** Distance distribution analyzed as described in the methods. The light color bands represent confidence bands. **d** Distance distribution from DEER (solid lines) compared with predicted distribution from cryo-EM structures (dashed lines).

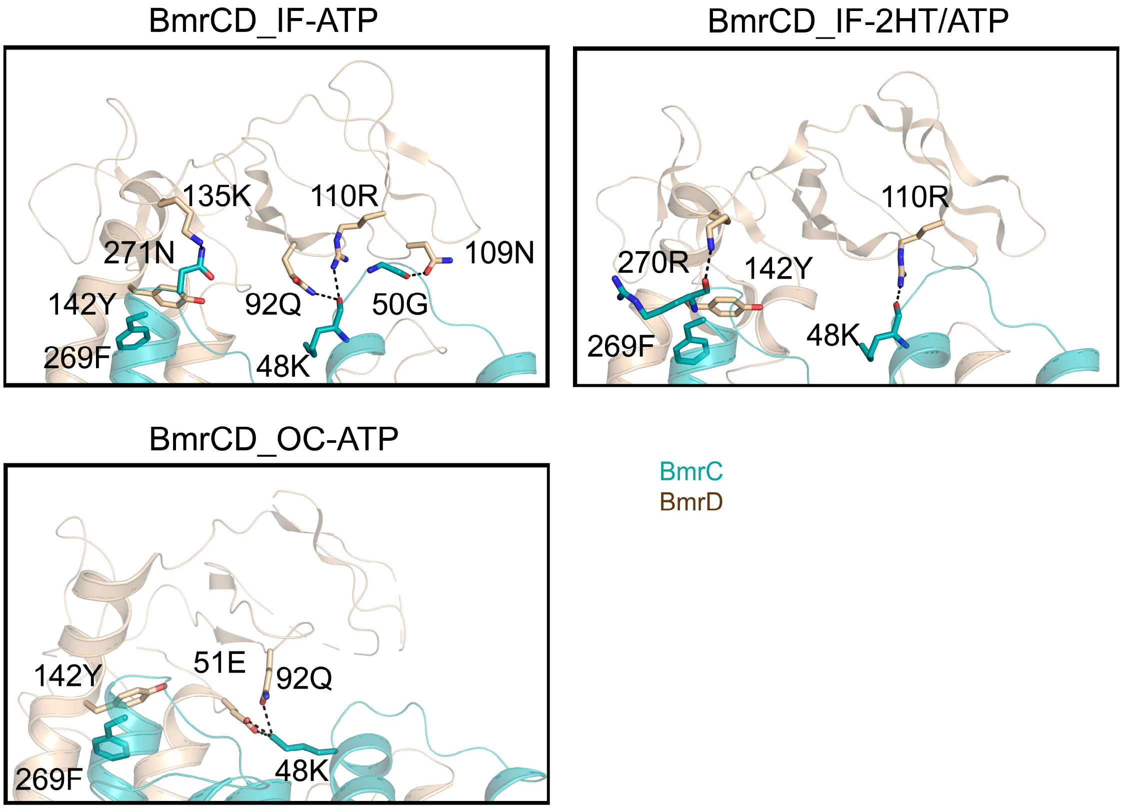

Supplementary Fig. 22: Interactions between the ECD and TMD.

Interactions between ECD and TMD of BmrCD_IF-2HT/ATP, BmrCD_IF-ATP BmrCD_OC-ATP. The sidechains of the interacting residues are represented by stick. Hydrogen bonds are shown in dashed line.

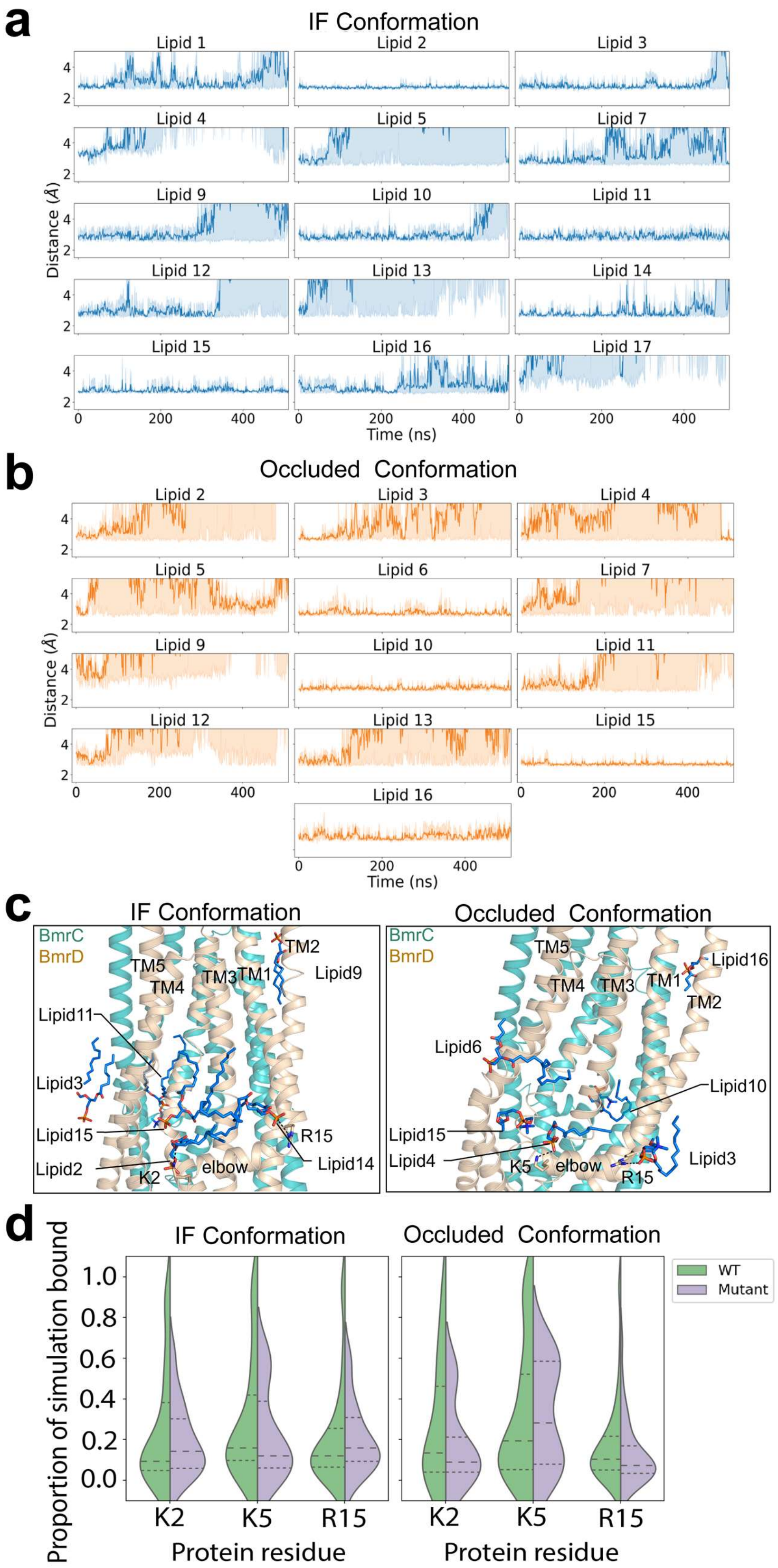

Supplementary Fig. 23: Assessment of lipid binding by MD simulations.

a and **b** Molecular dynamics (MD) simulation results of modeled lipids on BmrCD_IF-2HT/ATP (**a**) and BmrCD_OC-ATP (**b**), respectively. Minimum distance between modeled lipids and protein averaged across all simulation replicas with error bars shown in light blue. These plots highlight stably bound lipids which preserve their position with respect to the protein across all simulations for each protein conformation.
c Close-up view of TMDs shows stably binding lipids confirmed by the MD simulations. Lipids are shown in sticks with labeling. Possible interaction residues of BmrCD are represented in sticks, and hydrogen bonds are shown as dashed lines. **d** Lipid binding residence time is shown as densities for both WT and mutant BmrCD systems. Comparison of these densities demonstrates a decrease in high-residence lipid binding for any of the BmrD elbow helix mutants K2A, K5A and R15A whose positions are shown in (**c**).

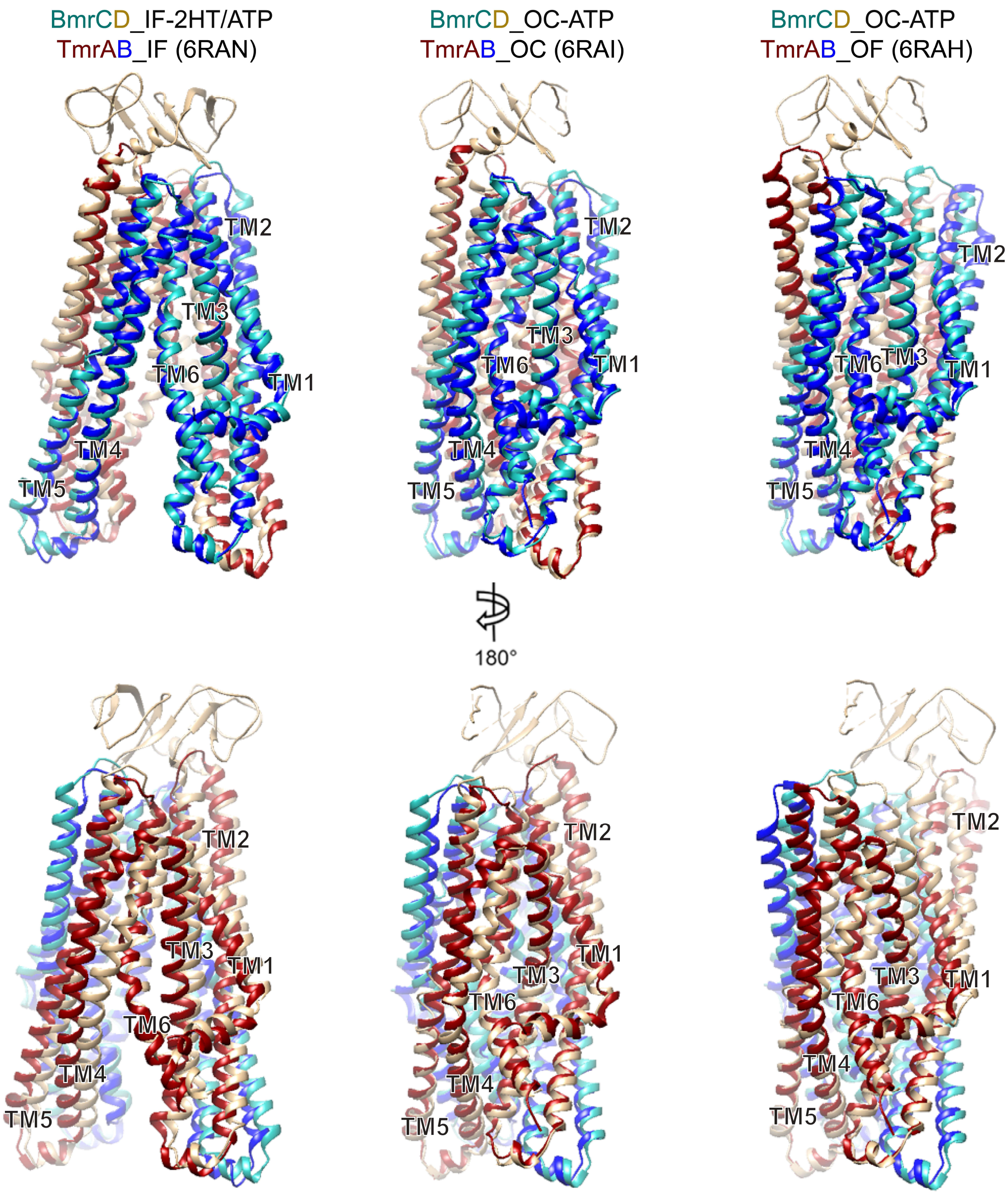

Supplementary Fig. 24: BmrCD compared to TmrAB in different conformations. BmrCD and TmrAB are represented by cartoon and superposed. Three structures of TmrAB are used for comparison: 6RAN (https://www.rcsb.org/structure/6RAN), 6RAI (https://www.rcsb.org/structure/6RAI) and 6RAH (https://www.rcsb.org/structure/6RAH).

References

1. Laskowski, R.A. & Swindells, M.B. LigPlot+: multiple ligand-protein interaction diagrams for drug discovery. *J Chem Inf Model* **51**, 2778-86 (2011).
2. Robert, X. & Gouet, P. Deciphering key features in protein structures with the new ENDscript server. *Nucleic Acids Res* **42**, W320-4 (2014).
