# Supplementary material for: Asymmetric conformations and lipid interactions shape the ATP-coupled cycle of a heterodimeric ABC transporter
Source: Nat Commun. 2023 Nov 8;14:7184. doi: 10.1038/s41467-023-42937-5 (PMC10632425; doi:10.1038/s41467-023-42937-5)
Supplement: Supplementary file 3 — Description of Additional Supplementary Files [file 41467_2023_42937_MOESM3_ESM.pdf]

File Name: Supplementary Data 1

Description: Analysis demonstrating the convergence of MD simulations and details about the MD simulation system setups.

File Name: Supplementary Data 2

Description: The PDB and PSF files for the initial and final structures in BmrCD IF conformation simulations, as well as sample configuration files for equilibrium and production, and the parameter files used in BmrCD IF MD simulations.

File Name: Supplementary Data 3

Description: The PDB and PSF files for the initial and final structures in BmrCD occluded conformation simulations, as well as sample configuration files for equilibrium and production, and the parameter files used in BmrCD occluded MD simulations.
