# Supplementary material for: Asymmetric conformations and lipid interactions shape the ATP-coupled cycle of a heterodimeric ABC transporter
Source: Nat Commun. 2023 Nov 8;14:7184. doi: 10.1038/s41467-023-42937-5 (PMC10632425; doi:10.1038/s41467-023-42937-5)
Supplement: Supplementary file 4 — Supplementary Data 1 [file 41467_2023_42937_MOESM4_ESM.docx]

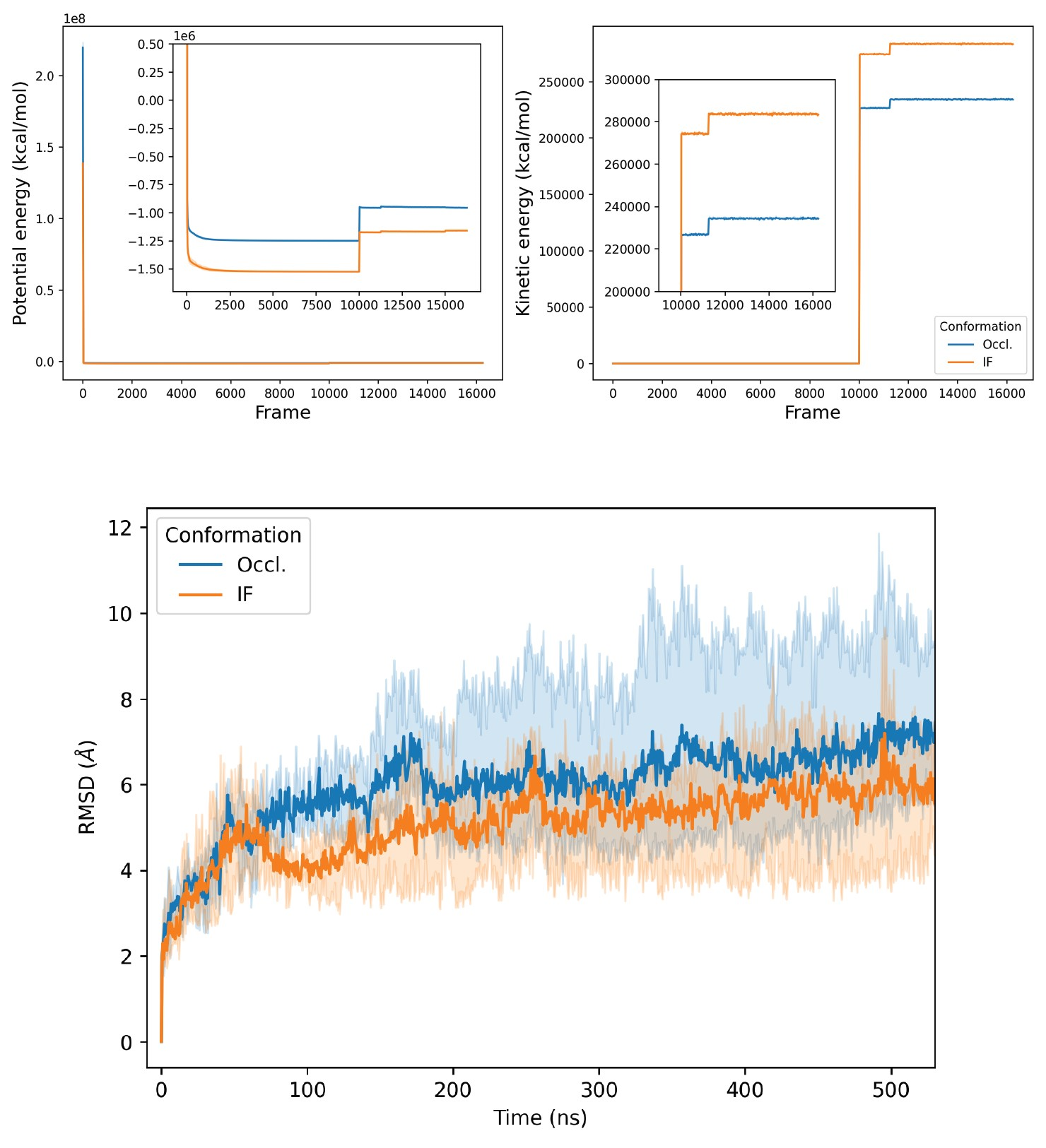


**Fig. 1**  **Convergence of simulations and analysis.**

The upper panels illustrate the equilibration of both potential and kinetic energies within both conformations of BmrCD systems. The lower panel illustrates the RMSD trace during the simulations, indicating that RMSD values stabilize after 100 ns.

**Table 1: Details about the MD simulation system setups.**

| **System name** | **IF conformation** | **Occluded conformation** |
| --- | --- | --- |
| **Simulation box dimensions (Å^3^)** | 176 Å x173 Å x192 Å | 150 Å x151 Å x189 Å |
| **Total number of atoms** | $\sim$439,000 | $\sim$352,000 |
| **Total number of water molecules** | $\sim$110,000 | $\sim$87,000 |
| **Salt concentration** | 0.15 M NaCl | 0.15 M NaCl |
| **Lipid composition** | 9:1 POPC:POPA | 9:1 POPC:POPA |
| **Replicas** | 3 | 3 |
| **Simulation files** | MD Supplementary Data 2 (simulation_files_IF_conformation.zip) | MD Supplementary Data 3  (simulation_files_OCC_conformation.zip) |
