# Supplementary material for: Asymmetric conformations and lipid interactions shape the ATP-coupled cycle of a heterodimeric ABC transporter
Source: Nat Commun. 2023 Nov 8;14:7184. doi: 10.1038/s41467-023-42937-5 (PMC10632425; doi:10.1038/s41467-023-42937-5)
Supplement: Supplementary file 7 — Reporting Summary [file 41467_2023_42937_MOESM7_ESM.pdf]

nature portfolio

Corresponding author(s): Hassane S. Mchaourab

Last updated by author(s): 10/14/2023

Reporting Summary

Nature Portfolio wishes to improve the reproducibility of the work that we publish. This form provides structure for consistency and transparency in reporting. For further information on Nature Portfolio policies, see our Editorial Policies and the Editorial Policy Checklist.

Statistics

For all statistical analyses, confirm that the following items are present in the figure legend, table legend, main text, or Methods section.

n/a	Confirmed	
☐	☒	The exact sample size (*n*) for each experimental group/condition, given as a discrete number and unit of measurement
☒	☐	A statement on whether measurements were taken from distinct samples or whether the same sample was measured repeatedly
☒	☐	The statistical test(s) used AND whether they are one- or two-sided<br>*Only common tests should be described solely by name; describe more complex techniques in the Methods section.*
☒	☐	A description of all covariates tested
☒	☐	A description of any assumptions or corrections, such as tests of normality and adjustment for multiple comparisons
☐	☒	A full description of the statistical parameters including central tendency (e.g. means) or other basic estimates (e.g. regression coefficient) AND variation (e.g. standard deviation) or associated estimates of uncertainty (e.g. confidence intervals)
☒	☐	For null hypothesis testing, the test statistic (e.g. *F*, *t*, *r*) with confidence intervals, effect sizes, degrees of freedom and *P* value noted<br>*Give P values as exact values whenever suitable.*
☒	☐	For Bayesian analysis, information on the choice of priors and Markov chain Monte Carlo settings
☒	☐	For hierarchical and complex designs, identification of the appropriate level for tests and full reporting of outcomes
☒	☐	Estimates of effect sizes (e.g. Cohen's *d*, Pearson's *r*), indicating how they were calculated

Our web collection on statistics for biologists contains articles on many of the points above.

Software and code

Policy information about availability of computer code

Data collection	EPU V2.10, Xepr ver 2.8b.1, NAMD 2.14, CHARMM36m and CHARMM36 force fields
Data analysis	Relion 3.1, MotionCor2 (1.3.1), Gtcf (1.0.6), cryoSPARC v3.2.0 and v4.1.2, phenix-1.20.1-4887, coot v0.9.8.8 EL (ccp4), pymol v2.5.4, chimera 1.5, chimeraX v1.3, DeerA, VMD, SciPy/Seaborn, CHARMM-GUI web server, MOE (Chemical Computing Group, Montreal, Canada), VMD.

For manuscripts utilizing custom algorithms or software that are central to the research but not yet described in published literature, software must be made available to editors and reviewers. We strongly encourage code deposition in a community repository (e.g. GitHub). See the Nature Portfolio guidelines for submitting code & software for further information.

Data

Policy information about availability of data

All manuscripts must include a data availability statement. This statement should provide the following information, where applicable:

- Accession codes, unique identifiers, or web links for publicly available datasets
- A description of any restrictions on data availability
- For clinical datasets or third party data, please ensure that the statement adheres to our policy

The cryo-EM maps of the heterodimeric ABC transporter BmrCD in nanodiscs have been deposited in the Electron Microscopy Data Bank (EMDB) under accession codes EMD-29297 [https://www.ebi.ac.uk/pdbe/entry/emdb/EMD-29297] (BmrCD_IF-2H/ATP); EMD-40908 [https://www.ebi.ac.uk/pdbe/entry/emdb/EMD-40908] (BmrCD_IF-H/ATP); EMD-29362 [https://www.ebi.ac.uk/pdbe/entry/emdb/EMD-29362] (BmrCD_IF-ATP); EMD-41004 [https://www.ebi.ac.uk/pdbe/entry/emdb/EMD-41004] (BmrCD_IF-ATP2); EMD-29087 [https://www.ebi.ac.uk/pdbe/entry/emdb/EMD-29087] (BmrCD_OC-ATP); EMD-40974 [https://www.ebi.ac.uk/pdbe/

entry/emdb/EMD-40974] (BmrCD_OC-ADPVi); EMD-41058 [https://www.ebi.ac.uk/pdbe/entry/emdb/EMD- 41085] (BmrCD_IF-H/ADPVi); The atomic coordinates have been deposited in the Protein Data Bank (PDB) under accession codes PDB-8FMV [https://doi.org/10.2210/pdb 8FMV/pdb] (BmrCD_IF-2H/ATP); PDB-8SZC [https://doi.org/10.2210/pdb 8SZC/pdb] (BmrCD_IF-H/ATP); PDB- 8FPF [https://doi.org/10.2210/pdb 8FPF/pdb] (BmrCD_IF-ATP); PDB-8T3K [https://doi.org/10.2210/pdb 8T3K/pdb] (BmrCD_IF-ATP2); PDB-8FHK [https://doi.org/10.2210/pdb 8FHK/pdb] (BmrCD_OC-ATP); PDB-8T1P [https://doi.org/10.2210/pdb 8T1P/pdb] (BmrCD_OC-ADPVi). For BmrCD_IF-H/ADPVi, no PDB structure deposited due to low resolution.
One published BmrCD detergent structure (PDB ID: 7M33) was used for comparison, and its map (EMDB ID: EMD-23641) was used for initial model. Three TmrAB structures used for comparison were TmrAB_IF (PDB ID: 6RAN), TmrAB_OC (PDB ID: 6RAI), and TmrAB_OF (PDB ID: 6RAH).

Research involving human participants, their data, or biological material

Policy information about studies with human participants or human data. See also policy information about sex, gender (identity/presentation), and sexual orientation and race, ethnicity and racism.

Reporting on sex and gender	N/A
Reporting on race, ethnicity, or other socially relevant groupings	N/A
Population characteristics	N/A
Recruitment	N/A
Ethics oversight	N/A

Note that full information on the approval of the study protocol must also be provided in the manuscript.

Field-specific reporting

Please select the one below that is the best fit for your research. If you are not sure, read the appropriate sections before making your selection.

[x] Life sciences   [ ] Behavioural & social sciences   [ ] Ecological, evolutionary & environmental sciences

For a reference copy of the document with all sections, see nature.com/documents/nr-reporting-summary-flat.pdf

Life sciences study design

All studies must disclose on these points even when the disclosure is negative.

Sample size	76,190 ~ 262,341 single particle images from 3722 ~ 12,706 micrographs were used for 3D reconstructions. No statistical methods were used to determine sample size for cryo-EM data.
Data exclusions	Bad particles were excluded based on the 2D and 3D classifications for pursuing high resolution maps. The particles of other conformations were excluded for pursuing high resolution for one conformation, for example, the particles of the inward-facing conformation were excluded for pursuing high resolution for the occluded conformation.
Replication	Datasets 1, 2, 3, and 4 were collected by four biological replications of BmrCD_CL-QQ sample.
Randomization	The datasets were randomly split in two halves which were refined independently. Resolution was assessed based on the Fourier shell correlation between the half maps at the 0.143 threshold criteria.
Blinding	Blinding is not applicable to this study since the samples investigated are required to be known.

Reporting for specific materials, systems and methods

We require information from authors about some types of materials, experimental systems and methods used in many studies. Here, indicate whether each material, system or method listed is relevant to your study. If you are not sure if a list item applies to your research, read the appropriate section before selecting a response.

Materials & experimental systems

n/a	Involved in the study
[x]	[ ] Antibodies
[x]	[ ] Eukaryotic cell lines
[x]	[ ] Palaeontology and archaeology
[x]	[ ] Animals and other organisms
[x]	[ ] Clinical data
[x]	[ ] Dual use research of concern
[x]	[ ] Plants

Methods

n/a	Involved in the study
[x]	[ ] ChIP-seq
[x]	[ ] Flow cytometry
[x]	[ ] MRI-based neuroimaging
